# Knowledge, attitudes, practice, and public health education demand regarding PARI prevention: a cross-sectional study among Chinese undergraduates

**DOI:** 10.3389/fpubh.2024.1387789

**Published:** 2024-06-20

**Authors:** Yuzhe Kong, Xiaoyi Zhu, Yang Yang, Haitao Xu, LingFeng Ma, Yu Zuo

**Affiliations:** ^1^Xiangya School of Medicine, Central South University, Changsha, Hunan, China; ^2^Xiangya School of Public Health, Central South University, Changsha, Hunan, China; ^3^Department of Prehospital Emergency, Third Xiangya Hospital of Central South University, Changsha, Hunan, China

**Keywords:** PARI, undergraduates, public health education, KAP, public health

## Abstract

**Objectives:**

The purpose of this study was to assess the level of knowledge, attitudes, and practices (KAP) of university students in China regarding the need for PARI and public health education.

**Methods:**

A cross-sectional online and offline survey was conducted in China website through Wenjuanxing and in different cities such as Changsha Hunan Province, Shanghai, Chongqing and in different public scenarios, such as hospitals, universities, and commercial venues between September 1 and September 7, 2023, using a 28-question questionnaire designed and reviewed by multidisciplinary experts.

**Results:**

A total of 4,096 respondents were recruited for this study, with 3,957 valid questionnaires. The mean knowledge score was 1.84 ± 0.52, the mean attitude score was 2.12 ± 0.51, and the mean practice score was 3.18 ± 0.55. Regression analyses found that: region, grade, school, and weekly anaerobic exercise time were influences on the knowledge score; region, grade, school, and weekly anaerobic exercise time were influences on the attitude score; region, grade, school attended, weekly anaerobic exercise time and weekly anaerobic exercise time as influences on the practice score. Subgroup analyses revealed that undergraduates from southern regions and 985 schools had higher knowledge attitude scores and lower practice scores. As the grade level increased, the knowledge and attitude scores showed a V-shaped trend and the behavior scores showed an inverted V-shaped trend. Correlation analysis found a positive correlation between knowledge and attitude scores, and a negative correlation between both and behavior, respectively. The public health education needs survey found that undergraduate students generally preferred guided instruction methods and content centered on the RICE principles, they preferred learning through books and pamphlets, and they were happy to see relevant content promoted in the campus environment.

**Conclusion:**

This study shows that Chinese undergraduate students have less knowledge, neutral attitudes, and good behaviors regarding PARI prevention. Special attention should be paid to meeting the needs of undergraduate students for public health education to equip them with relevant knowledge so that they can better behave in PARI prevention.

## 1 Introduction

Physical activity-related injuries (PARI) are becoming increasingly prevalent among Chinese undergraduates (1, 2), reflecting a broader trend observed in various physical activities worldwide. These injuries encompass a wide range, from minor sprains and strains to more severe cases like fractures and concussions (3). Such injuries often occur abruptly during sports or physical exercises. They not only impede the continuation of activities but can also lead to long-term health consequences (4).

In the context of China’s rapidly growing engagement in sports and physical activities, especially among university students, these injuries pose a significant challenge (4, 5). Adults and youth participating in these activities are susceptible to different types of injuries (6). Adults commonly report joint and muscle strains, while younger individuals might experience injuries unique to their developing bodies, such as growth plate injuries (7).

These incidents can substantially impact an individual’s daily life, leading to reduced physical activity levels and, consequently, affecting overall health and well-being (8). If not properly managed, PARI can evolve into chronic problems and heighten the risk of re-injury (9, 10).

Although the incidence of such injuries is increasing, several relevant studies conducted by previous investigators have found that there is a significant lack of awareness and understanding of the prevention of such injuries (5, 11, 12). Deficiencies in this area may result in inadequate PARI prevention and management strategies, exacerbating the risk of injury and hindering recovery (13–15).

However, there is a paucity of data with large-scale samples focused on China regarding the knowledge, attitude, and practice (KAP) regarding PARI prevention and public health education demand among undergraduates. The undergraduate phase may be accompanied by an increase in high-risk behaviors, leading to health issues such as sports injuries (2, 11, 13). Through research, it is possible to better understand the causes of these behaviors and develop effective prevention and intervention measures. Therefore, the purpose of this study is to assess the current KAP levels and to investigate the public health education needs of Chinese undergraduates for the prevention of PARI. In addition, we further explored the influencing factors for the current KAP level and the public health education demand help to determine whether there exist correlations between the demographic data and medical the current KAP level and the public health education demand, respectively.

## 2 Methods

### 2.1 Participants

Before distributing the survey, the minimum sample size was calculated using G*Power (version 3.1; Heinrich Heine University) to achieve a power of 0.80. In the G*Power software, a logistic regression test was conducted for *a priori* power calculation with an odds ratio (OR) of 1.2 and a significance level of 0.05. The minimum sample needed to achieve a power of 0.99 was 3,460 for our study. Considering the missing and non-responsive cases, we expanded it by 10%, yielding a predicted sample size of 3,806 (16).

In the present study, the population was undergraduate students enrolled in Chinese universities. Those who were unwilling to participate were not invited.

In the process of data screening, the inclusion criteria were: ① undergraduate students enrolled in Chinese universities; ② volunteered to participate; ③ competent to comprehend the content of the questionnaire; ④ signed informed consent form. The exclusion criteria were: ① those who answered contradictory or factual content; ② the response time was ≤80s.

### 2.2 Ethics approval

Ethical approval regarding human subject research was obtained from the Ethics Committee on Third Xiangya Hospital of Central South University (approval number: Fast24084). Informed consent was obtained from each participant online by placing a question about their agreement to participate in the study at the beginning of the survey. Participants were assured of the confidentiality and anonymity of this study and their rights to exit at any time. We declare that the data were collected for academic use only.

### 2.3 Instrument

The primary version of the questionnaire was developed in Chinese by an investigation team based on a deep literature review of comparable studies and international guidelines (17–19).

Researchers randomly invited 8 participants face-to-face from the general population to answer the questionnaire online for pretext and collected their feedback about the comprehensibility of questions and options.

Two experts in the field of PARI reviewed these responses and each item of the survey and confirmed the final version of a 28-item questionnaire (see Supplementary Material). It comprised basic demographic data and 4 sections about knowledge, attitudes, practices, and demand regarding popular healthcare toward sports health.

A series of options were listed with points for each question. The way in which the options correspond to the points is shown in Table 1. The total scores for knowledge, attitude and practice in PARI were 24. Cronbach’s a was >0.7 for each scale (0.823 for knowledge, 0.811 for attitude and 0.842 for practice).

**Table 1:** Scoring method.

Option	Point	Option	Point	Option	Point
Not at all	1	Strongly disagree	1	Seldom	1
Not clear	2	Disagree	2	Sometimes	2
Clear	3	Agree	3	Often	3
Completely clear	4	Strongly agree	4	Always	4

### 2.4 Procedures

**Figure 1:**
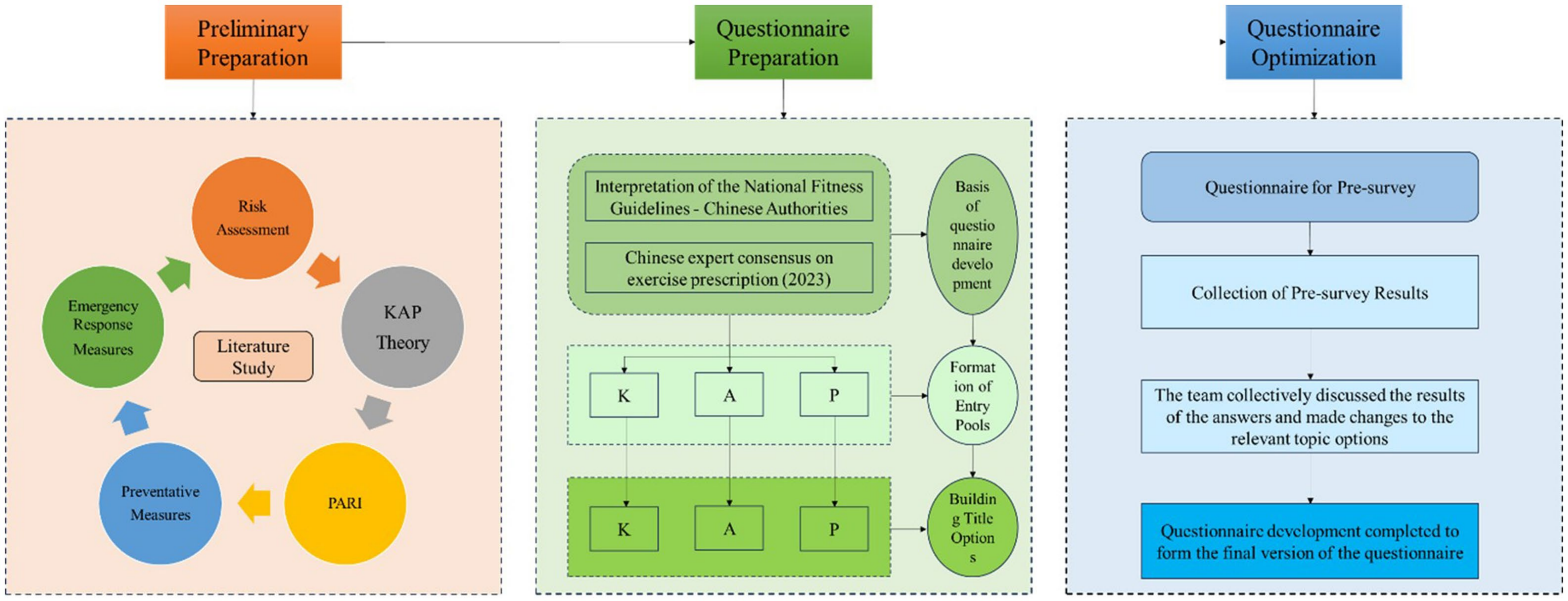
Questionnaire production flowchart.

The cross-sectional survey was conducted in China between 1st September and 7th September 2023, using a 28-item questionnaire designed and reviewed by multidisciplinary experts (see Figure 1).

The general Chinese adult population was randomly invited online and offline. Participants were informed that the survey was based on voluntary principles and that their data would be anonymous and confidential. First, our investigation team created a questionnaire QR code (quick response code) by Wenjuanxing,^1^ which is an online questionnaire platform widely used in academic studies in China. Then, researchers distributed the QR code using Chinese popular social media to get access to the general populations as many as possible, including WeChat and QQ. In addition, 3 researchers performed face-to-face invitations to scan the QR code in possible surveyed populations in different cities such as Changsha Hunan Province, Shanghai, Chongqing and in different public scenarios, such as hospitals, universities, and commercial venues.

Participants’ IP addresses were restricted to ensure only 1 submission. The chief researcher was responsible for checking the collected data from Wenjuanxing, and 3.39% of submitted questionnaires were excluded for invalid response times and logistic errors.

### 2.5 Main outcomes

The main outcomes include scores of each 3 parts: knowledge, attitude and practice and the answer to the questions of the public health education demand part.

### 2.6 Covariates

Gender, region, grade, school, duration of aerobic exercise per week and duration of anaerobic exercise per week.

We have divided China into six regions. Heilongjiang, Jilin, and Liaoning provinces belong to the northeast region of China. Inner Mongolia Autonomous Region, Shanxi Province, Hebei Province, Beijing Municipality, and Tianjin Municipality belong to the northern region of China. Shandong, Jiangsu, Shanghai, Zhejiang, Fujian, Anhui, and Jiangxi provinces belong to the eastern region of China. Henan, Hainan, Hubei, Hunan, Guangdong, Guangxi Zhuang Autonomous Region and Hainan belong to the south-central region of China. Tibet Autonomous Region, Sichuan Province, Yunnan Province, Chongqing Municipality, Guizhou Province belong to the Southwest region of China. Xinjiang Uygur Autonomous Region, Gansu Province, Qinghai Province, Ningxia Hui Autonomous Region, and Shaanxi Province belong to the northwestern region of China.

In China, we generally have a way of classifying schools into 985 project universities, 211 project universities, public universities, and private universities. Their comprehensive strength and student quality decrease in turn. Thus, in this study, we also use this type of classification to categorize the schools that the participants are in.

The team referred to the “Interpretation of the <National Fitness Guidelines>” and the “Expert Consensus on Exercise Prescription (2023)” (19) for the description of daily recommended exercise duration. The team decided to set the weekly aerobic exercise duration interval as 2 h, i.e., weekly aerobic exercise duration ≤2 h, >2 h and ≤4 h, >4 h and ≤6 h, and >6 h. In addition, the weekly anaerobic exercise duration interval was set to 1 h, i.e., weekly aerobic exercise duration ≤1 h, > 1 h and ≤2 h, >2 h and ≤3 h, and >3 h.

### 2.7 Statistical analysis

Data were summarized as means and standard deviations for continuous variables and percentages for categorical variables. Comparisons between groups were made using t-tests and chi-square tests. ANOVA analysis, multivariate logistic regression model and multiple linear regression model was used to assess the association between various demographic factors and KAP levels and public health educational needs. The relationship between KAP was determined using Pearson correlation. Also, subgroup analyses will be conducted to further assess the stability of the associations between KAP by subgroups of gender, age, location, school location, grade level, aerobic exercise time, and anaerobic exercise time. All statistical analyses were performed using R 4.3.2 and two-sided tests with a significance level of 5% (*p* < 0.05).

## 3 Results

### 3.1 Baseline characteristics of individuals

The study surveyed 3,957 individuals (see Table 2). The gender distribution was nearly equal with 1,971 males (49.81%) and 1,986 females (50.19%). Participants were from various regions of China (see Supplementary Figure S1), with the majority from East China (56.79%), followed by South Central China (17.03%), Northeast China (8.24%), North China (6.75%), Northwest China (6.47%), and Southwest China (4.73%). The respondents were spread across academic grades, with 21.51% freshmen, 30.40% sophomores, 29.59% juniors, and 18.50% seniors. Regarding their university type, 15.31% attended Project 985 universities, 13.77% were from Project 211 universities, 46.78% were from state universities, and 24.13% from private universities. For aerobic exercise, 54.36% exercised less than 2 h per week, 27.82% for 2 to 4 h, 11.55% for 4 to 6 h, and 6.27% for more than 6 h. For anaerobic exercise, 28.38% exercised less than 1 h per week, 33.13% for 1 to 2 h, 26.91% for 2 to 3 h, and 11.57% for more than 3 h per week.

**Table 2:** Demographics.

Item		Data	
		*n*	%
Population		3,957	/
Gender	Male	1,971	49.81
	Female	1,986	50.19
Region	East China	2,247	56.79
	North China	267	6.75
	Northeast China	326	8.24
	Northwest China	256	6.47
	South Central China	674	17.03
	South West China	187	4.73
Grade	Freshman	851	21.51
	Sophomore	1,203	30.40
	Junior	1,171	29.59
	Senior	732	18.50
School	Project 985 University	606	15.31
	Project 211 University	545	13.77
	State University	1,851	46.78
	Private University	955	24.13
Duration of aerobic exercise per week	Less than 2 h	2,151	54.36
	2 ~ 4 h	1,101	27.82
	4 ~ 6 h	457	11.55
	More than 6 h	248	6.27
Duration of anaerobic exercise per week	Less than 1 h	1,123	28.38
	1 ~ 2 h	1,311	33.13
	2 ~ 3 h	1,065	26.91
	More than 3 h	458	11.57

### 3.2 Knowledge of PARI prevention

The average score for the knowledge section was 1.84 ± 0.52. Upon examining the scores of each section, it was observed that the PARI risk assessment (1.84 ± 0.69) mirrored the overall level of knowledge, whereas familiarity with PARI preventative measures was higher (1.88 ± 0.67), and knowledge regarding PARI emergency response measures was comparatively lower (1.81 ± 0.90). Subsequent subgroup analysis (see Table 3) revealed that scores were higher in the Central and Southern regions (1.98 ± 0.61), and the level of knowledge demonstrated a “V-shaped” trend with advancing grades. Additionally, it was discovered that respondents from Project 985 universities (2.3 ± 0.7) and those who engaged in less than 1 h of anaerobic exercise weekly (2.01 ± 0.62) scored higher.

**Table 3:** Knowledge scores by subgroup.

First-level items	Second-level items	Total		PARI risk assessment		PARI preventive measures		PARI emergency measures	
Overall		1.84 ± 0.52	1.72(1.44,2.11)	1.84 ± 0.69	1.50(1.50,2.50)	1.88 ± 0.67	1.67(1.33,2.33)	1.81 ± 0.90	2.00(1.00,2.00)
Gender	Male	1.85 ± 0.52	1.67(1.50,2.00)	1.83 ± 0.68	1.50(1.50,2.50)	1.87 ± 0.67	1.67(1.33,2.33)	1.79 ± 0.88	2.00(1.00,2.00)
	Female	1.86 ± 0.52	1.83(1.50,2.17)	1.85 ± 0.69	1.50(1.50,2.50)	1.89 ± 0.67	1.67(1.33,2.33)	1.82 ± 0.92	2.00(1.00,2.00)
Region	Northeast China	1.77 ± 0.46	1.72(1.44,2.04)	1.78 ± 0.66	1.50(1.50,2.00)	1.79 ± 0.60	1.67(1.33,2.00)	1.74 ± 0.91	1.00(1.00,2.00)
	North China	1.85 ± 0.57	1.72(1.44,2.11)	1.85 ± 0.74	1.50(1.25,2.50)	1.95 ± 0.72	1.67(1.33,2.33)	1.76 ± 0.89	2.00(1.00,2.00)
	East China	1.8 ± 0.48	1.72(1.44,2.06)	1.80 ± 0.66	1.50(1.50,2.00)	1.82 ± 0.61	1.67(1.33,2.00)	1.79 ± 0.90	2.00(1.00,2.00)
	South Central China	1.98 ± 0.61	1.83(1.50,2.39)	1.96 ± 0.72	2.00(1.50,2.50)	2.08 ± 0.80	2.00(1.33,2.67)	1.90 ± 0.91	2.00(1.00,2.00)
	Southwest China	1.88 ± 0.56	1.78(1.50,2.17)	1.87 ± 0.72	1.50(1.50,2.50)	1.97 ± 0.70	2.00(1.33,2.33)	1.81 ± 0.87	2.00(1.00,2.00)
	Northwest China	1.89 ± 0.54	1.78(1.44,2.22)	1.9 0 ± 0.71	2.00(1.50,2.50)	1.91 ± 0.70	1.67(1.33,2.33)	1.86 ± 0.91	2.00(1.00,2.00)
Grade	Grade 1	1.93 ± 0.56	1.83(1.50,2.28)	1.92 ± 0.73	2.00(1.50,2.50)	2.01 ± 0.75	2.00(1.33,2.33)	1.85 ± 0.90	2.00(1.00,2.00)
	Grade 2	1.87 ± 0.55	1.78(1.44,2.17)	1.85 ± 0.66	1.50(1.50,2.50)	1.91 ± 0.70	1.67(1.33,2.33)	1.84 ± 0.92	2.00(1.00,2.00)
	Grade 3	1.76 ± 0.45	1.72(1.44,2.00)	1.76 ± 0.65	1.50(1.00,2.00)	1.76 ± 0.55	1.67(1.33,2.00)	1.76 ± 0.88	2.00(1.00,2.00)
	Grade 4	1.82 ± 0.52	1.72(1.44,2.06)	1.85 ± 0.70	1.50(1.50,2.50)	1.85 ± 0.65	1.67(1.33,2.33)	1.77 ± 0.90	2.00(1.00,2.00)
	Grade 5	1.90 ± 0.59	1.78(1.50,2.11)	1.92 ± 0.70	2.00(1.50,2.00)	1.99 ± 0.74	2.00(1.33,2.33)	1.78 ± 0.90	2.00(1.00,2.00)
School	Project 985 University	2.30 ± 0.70	2.33(1.72,2.83)	2.26 ± 0.76	2.50(1.50,3.00)	2.52 ± 0.88	2.67(1.67,3.00)	2.13 ± 0.93	2.00(1.00,3.00)
	Project 211 University	1.81 ± 0.47	1.72(1.50,2.06)	1.82 ± 0.66	1.50(1.50,2.50)	1.83 ± 0.60	1.67(1.33,2.00)	1.77 ± 0.88	2.00(1.00,2.00)
	State University	1.75 ± 0.43	1.72(1.44,2.00)	1.75 ± 0.64	1.50(1.50,2.00)	1.76 ± 0.56	1.67(1.33,2.00)	1.75 ± 0.88	2.00(1.00,2.00)
	Private University	1.74 ± 0.43	1.67(1.42,2.00)	1.75 ± 0.64	1.50(1.00,2.00)	1.74 ± 0.52	1.67(1.33,2.00)	1.73 ± 0.89	1.00(1.00,2.00)
Duration of aerobic exercise per week	Less than 2 h	1.85 ± 0.53	1.78(1.44,2.17)	1.85 ± 0.69	1.50(1.50,2.50)	1.90 ± 0.68	1.67(1.33,2.33)	1.80 ± 0.89	2.00(1.00,2.00)
	2 ~ 4 h	1.84 ± 0.52	1.72(1.44,2.11)	1.83 ± 0.68	1.50(1.50,2.50)	1.88 ± 0.66	1.67(1.33,2.33)	1.81 ± 0.90	2.00(1.00,2.00)
	4 ~ 6 h	1.82 ± 0.51	1.72(1.44,2.06)	1.82 ± 0.68	1.50(1.50,2.50)	1.85 ± 0.65	1.67(1.33,2.00)	1.80 ± 0.93	2.00(1.00,2.00)
	More than 6 h	1.80 ± 0.55	1.72(1.44,2.00)	1.81 ± 0.69	1.50(1.50,2.00)	1.79 ± 0.68	1.67(1.33,2.00)	1.81 ± 0.90	2.00(1.00,2.00)
Duration of anaerobic exercise per week	Less than 1 h	2.01 ± 0.62	1.89(1.56,2.44)	1.99 ± 0.73	2.00(1.50,2.50)	2.14 ± 0.79	2.00(1.33,3.00)	1.91 ± 0.92	2.00(1.00,2.00)
	1 ~ 2 h	1.78 ± 0.47	1.72(1.44,2.06)	1.77 ± 0.65	1.50(1.50,2.00)	1.78 ± 0.59	1.67(1.33,2.00)	1.78 ± 0.90	2.00(1.00,2.00)
	2 ~ 3 h	1.76 ± 0.44	1.72(1.44,2.00)	1.79 ± 0.66	1.50(1.50,2.00)	1.75 ± 0.55	1.67(1.33,2.00)	1.74 ± 0.87	2.00(1.00,2.00)
	More than 3 h	1.80 ± 0.50	1.72(1.44,2.04)	1.81 ± 0.70	1.50(1.00,2.00)	1.82 ± 0.65	1.67(1.33,2.00)	1.77 ± 0.90	2.00(1.00,2.00)

Following this, we explored the factors influencing the overall score and the scores of the 3 sections. The results of the univariate analysis (see Supplementary Table S1) indicated that region, grade, school, and weekly duration of anaerobic exercise were significant factors influencing the overall score (*P* < 0.001); these same factors also affected the scores for PARI risk assessment (*P* < 0.001), PARI preventative measures (*P* < 0.001), and PARI emergency response measures (region: *P* < 0.05; school, and weekly duration of anaerobic exercise: *P* < 0.001). To eliminate the inter-correlation among independent variables, a multivariate linear regression analysis was conducted, which excluded the influence of grade on PARI risk assessment and the influence of region on PARI emergency response measures (see Supplementary Table S2).

**Figure 2:**
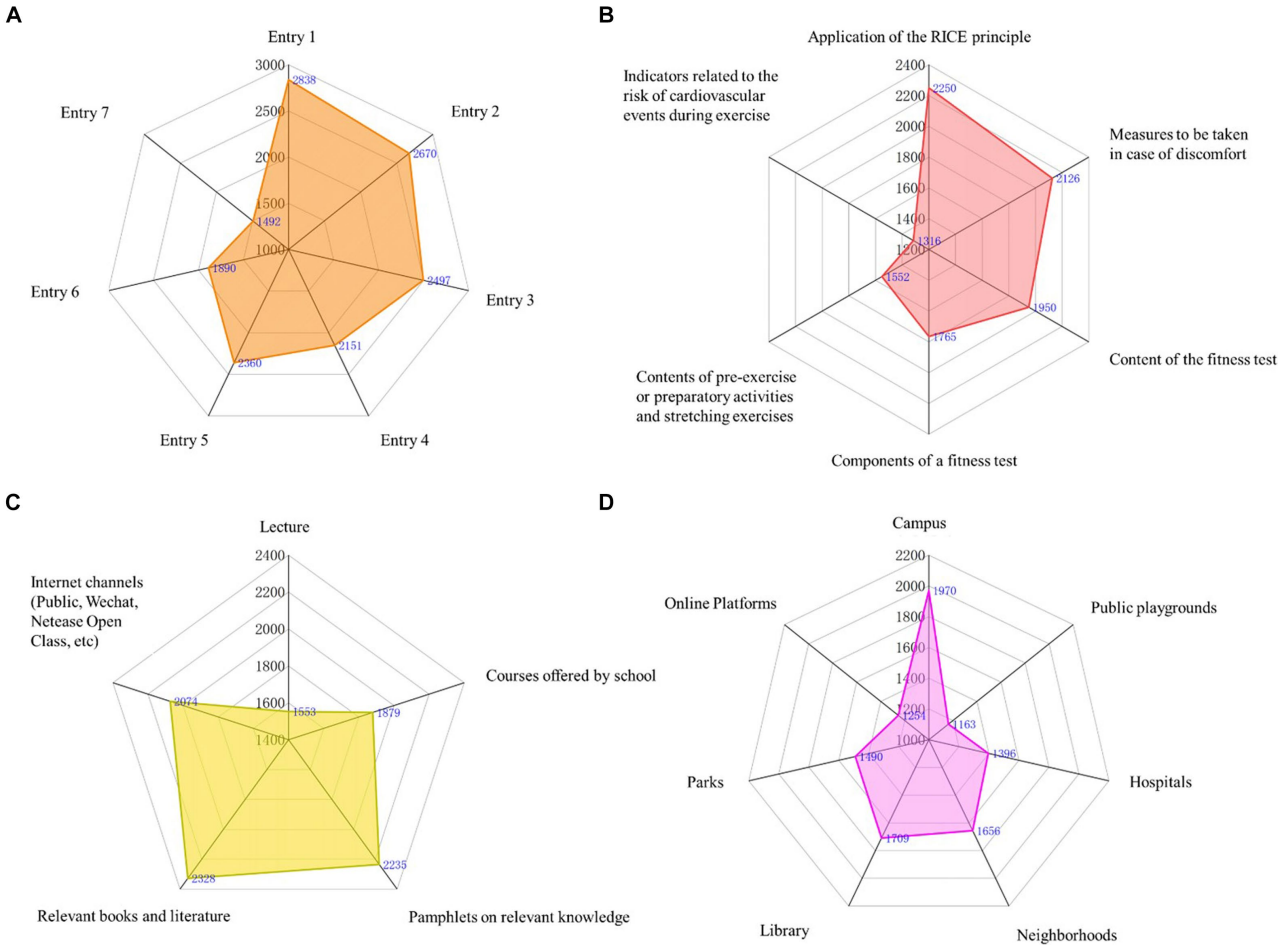
Number of people with each option for each question by subgroup.

Finally, focusing on each question, it was found that the majority of respondents were unaware of the answers (choosing “completely unaware” or “unaware”) for most questions, exceeding 75%. The percentage of respondents selecting each option for every question is shown in Supplementary Figure S2. Subsequently, we analyzed the selection frequency of each option for every question in different subgroups and found the responses to be similar to the overall trend, as shown in Figure 2.

### 3.3 Attitude of PARI prevention

The average score for the attitude section was 2.12 ± 0.51. Observing the scores across different sections, the PARI risk assessment (2.11 ± 0.66), PARI preventative measures (2.14 ± 0.61), and PARI emergency response measures (2.10 ± 0.87) were consistent with the overall attitude. Subsequent subgroup analysis (see Table 4) revealed more positive attitudes in the Central and Southern (2.29 ± 0.61) and Southwestern regions (2.22 ± 0.58), with a “V-shaped” trend in attitudes corresponding with ascending grade levels. Additionally, respondents from Project 985 universities (2.59 ± 0.69) and those engaging in less than 1 h of anaerobic exercise weekly (2.28 ± 0.64) demonstrated more positive attitudes.

**Table 4:** Attitude scores by subgroup.

First-level items	Second-level items	Total		PARI risk assessment		PARI preventive measures		PARI emergency measures	
Overall		2.12 ± 0.51	2(1.78,2.33)	2.11 ± 0.66	2(1.5,2.5)	2.14 ± 0.61	2(1.67,2.33)	2.1 ± 0.87	2(2,3)
Gender	Male	2.13 ± 0.48	2(1.83,2.33)	2.11 ± 0.65	2(1.5,2.5)	2.14 ± 0.61	2(1.67,2.5)	2.11 ± 0.86	2(2,3)
	Female	2.12 ± 0.51	2(1.83,2.33)	2.1 ± 0.67	2(1.5,2.5)	2.13 ± 0.61	2(1.67,2.33)	2.1 ± 0.88	2(1,3)
Region	Northeast China	2.01 ± 0.4	1.94(1.72,2.22)	1.98 ± 0.64	2(1.5,2.5)	2.02 ± 0.49	2(1.67,2.33)	2.02 ± 0.81	2(2,2)
	North China	2.16 ± 0.58	2(1.78,2.39)	2.13 ± 0.69	2(1.5,2.5)	2.11 ± 0.65	2(1.67,2.33)	2.24 ± 0.94	2(2,3)
	East China	2.06 ± 0.46	2(1.78,2.28)	2.07 ± 0.63	2(1.5,2.5)	2.08 ± 0.56	2(1.67,2.33)	2.04 ± 0.85	2(1,2)
	South Central China	2.29 ± 0.61	2.11(1.89,2.67)	2.24 ± 0.71	2(1.5,2.62)	2.35 ± 0.7	2.33(1.67,3)	2.27 ± 0.89	2(2,3)
	Southwest China	2.22 ± 0.58	2.11(1.83,2.56)	2.24 ± 0.67	2(1.5,2.5)	2.26 ± 0.7	2(1.67,2.67)	2.17 ± 0.86	2(2,3)
	Northwest China	2.12 ± 0.51	2(1.78,2.33)	2.11 ± 0.67	2(1.5,2.5)	2.14 ± 0.6	2(1.67,2.33)	2.11 ± 0.85	2(2,3)
Grade	Grade 1	2.22 ± 0.61	2.06(1.78,2.5)	2.19 ± 0.72	2(1.5,2.5)	2.24 ± 0.7	2(1.67,2.67)	2.22 ± 0.92	2(2,3)
	Grade 2	2.14 ± 0.52	2.06(1.78,2.39)	2.16 ± 0.66	2(1.5,2.5)	2.16 ± 0.63	2(1.67,2.67)	2.11 ± 0.85	2(2,3)
	Grade 3	2.03 ± 0.43	2(1.72,2.28)	2.02 ± 0.61	2(1.5,2.5)	2.05 ± 0.51	2(1.67,2.33)	2.02 ± 0.84	2(1,2)
	Grade 4	2.08 ± 0.45	2(1.78,2.33)	2.05 ± 0.61	2(1.5,2.5)	2.11 ± 0.58	2(1.67,2.33)	2.09 ± 0.84	2(2,2)
	Grade 5	2.14 ± 0.55	2(1.83,2.33)	2.08 ± 0.69	2(1.5,2.5)	2.18 ± 0.63	2(1.67,2.67)	2.16 ± 0.89	2(2,3)
School	Project 985 University	2.59 ± 0.69	2.61(2.01,3)	2.53 ± 0.76	2.5(2,3)	2.73 ± 0.74	3(2,3)	2.52 ± 0.92	3(2,3)
	Project 211 University	2.07 ± 0.48	2(1.78,2.28)	2.06 ± 0.64	2(1.5,2.5)	2.09 ± 0.57	2(1.67,2.33)	2.07 ± 0.85	2(2,2)
	State University	2.03 ± 0.41	2(1.72,2.28)	2.02 ± 0.61	2(1.5,2.5)	2.03 ± 0.51	2(1.67,2.33)	2.04 ± 0.83	2(1,2)
	Private University	2.01 ± 0.4	2(1.72,2.22)	2.03 ± 0.6	2(1.5,2.5)	2 ± 0.5	2(1.67,2.33)	1.99 ± 0.83	2(1,2)
Duration of aerobic exercise per week	Less than 2 h	2.13 ± 0.52	2.06(1.78,2.36)	2.13 ± 0.67	2(1.5,2.5)	2.15 ± 0.62	2(1.67,2.67)	2.1 ± 0.86	2(2,3)
	2 ~ 4 h	2.11 ± 0.5	2(1.78,2.33)	2.1 ± 0.65	2(1.5,2.5)	2.13 ± 0.61	2(1.67,2.33)	2.1 ± 0.85	2(2,3)
	4 ~ 6 h	2.09 ± 0.49	2(1.78,2.33)	2.05 ± 0.63	2(1.5,2.5)	2.1 ± 0.57	2(1.67,2.33)	2.12 ± 0.89	2(2,3)
	More than 6 h	2.1 ± 0.52	2(1.78,2.33)	2.06 ± 0.65	2(1.5,2.5)	2.12 ± 0.58	2(1.67,2.33)	2.14 ± 0.93	2(1,3)
Duration of anaerobic exercise per week	Less than 1 h	2.28 ± 0.64	2.17(1.83,2.72)	2.26 ± 0.73	2(1.5,3)	2.36 ± 0.71	2.33(1.67,3)	2.23 ± 0.91	2(2,3)
	1 ~ 2 h	2.06 ± 0.44	2(1.78,2.28)	2.06 ± 0.63	2(1.5,2.5)	2.06 ± 0.55	2(1.67,2.33)	2.06 ± 0.84	2(2,2)
	2 ~ 3 h	2.04 ± 0.41	2(1.78,2.28)	2.04 ± 0.6	2(1.5,2.5)	2.02 ± 0.5	2(1.67,2.33)	2.04 ± 0.85	2(1,2)
	More than 3 h	2.06 ± 0.48	2(1.72,2.22)	2.03 ± 0.64	2(1.5,2.5)	2.1 ± 0.59	2(1.67,2.33)	2.05 ± 0.84	2(2,2)

Further investigation into the factors influencing the overall and individual section scores was conducted. Univariate analysis results (see Supplementary Table S3) showed that region, grade, school, and weekly duration of anaerobic exercise were significant factors for the overall score (*P*<0.001), and similarly influenced the scores for PARI risk assessment (*P* < 0.001), PARI preventative measures (*P* < 0.001), and PARI emergency response measures (*P* < 0.001). Then, a multivariate linear regression analysis was undertaken, which eliminated the influence of region on PARI emergency response measures (see Supplementary Table S4).

**Figure 3:**
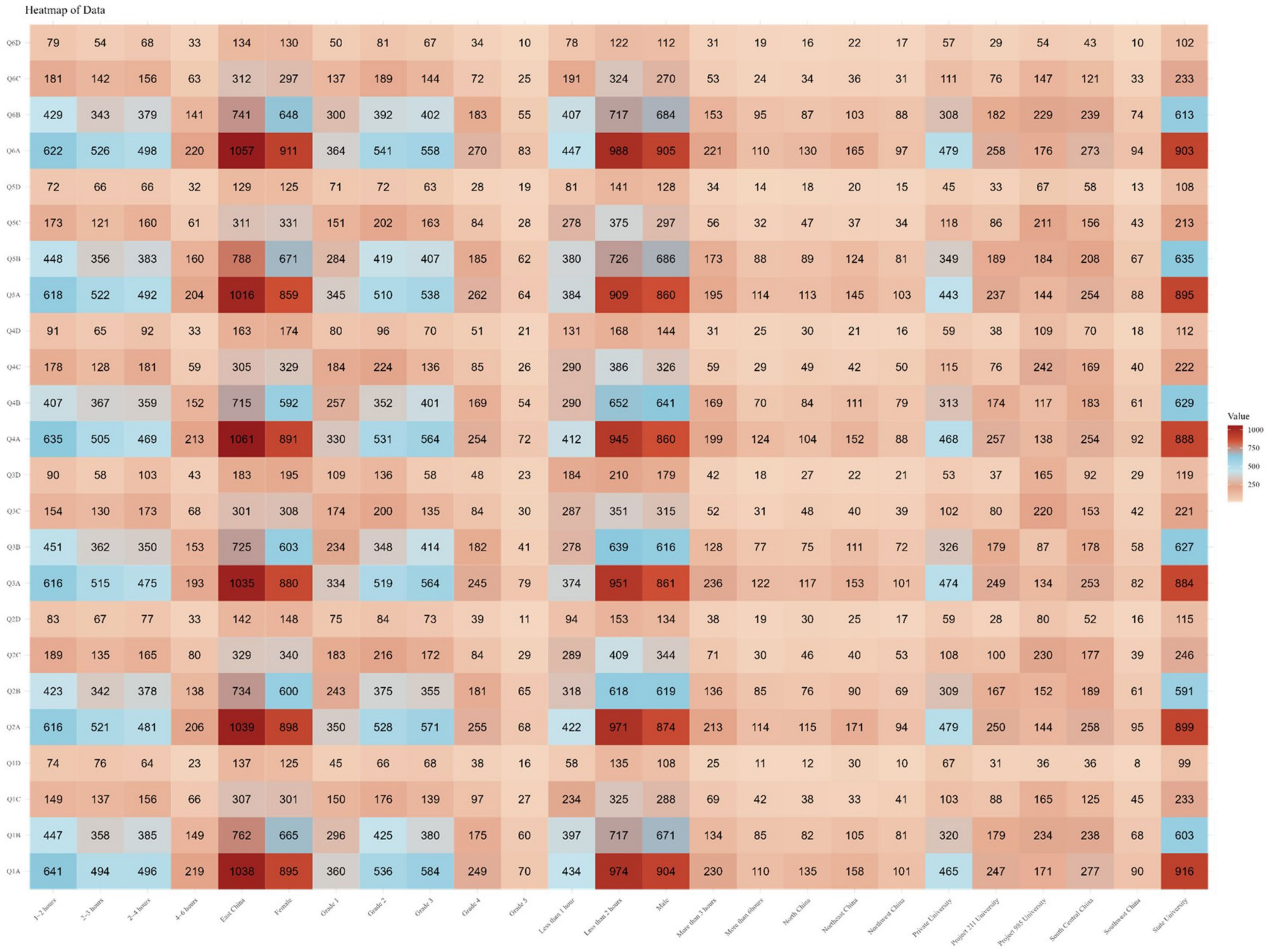
Number of people with each option for each question by subgroup.

Finally, focusing on each question, it was observed that the majority of respondents exhibited a neutral attitude (choosing either “disagree” or “agree”) toward each question, exceeding 65%. The percentage of respondents selecting each option for every question is illustrated in Supplementary Figure S3. Subsequently, the selection frequencies for each option of every question among different subgroups were analyzed, revealing that the response patterns were similar to the overall trend, as depicted in Figure 3.

### 3.4 Practice of PARI prevention

The average score for the practice section was 3.18 ± 0.55. Analyzing the scores across various components, it was found that the scores for PARI risk assessment (3.16 ± 0.78), PARI preventive measures (3.27 ± 0.56), and PARI emergency response measures (3.10 ± 1.04) were consistent with the overall practice pattern. Further subgroup analysis (see Table 5) revealed relatively poorer practices in the Central and Southern regions (3.03 ± 0.65) and the Southwestern regions (3.02 ± 0.66), with a trend in practice among respondents exhibiting an inverted “V-shape” as grades progressed. Additionally, it was noted that respondents from Project 985 universities (2.68 ± 0.74) and those engaging in less than 1 h of anaerobic exercise per week (2.95 ± 0.68) demonstrated poorer practices.

**Table 5:** Practice scores by subgroup.

First-level items	Second-level items	Total		PARI risk assessment		PARI preventive measures		PARI emergency measures	
Overall		3.18 ± 0.55	3.28(2.89,3.56)	3.16 ± 0.78	3.5(2.5,4)	3.27 ± 0.56	3.33(3,3.67)	3.1 ± 1.04	3(2,4)
Gender	Male	3.2 ± 0.49	3.33(3,3.5)	3.15 ± 0.78	3.5(2.5,4)	3.26 ± 0.56	3.33(3,3.67)	3.12 ± 1.02	3(2,4)
	Female	3.21 ± 0.49	3.33(3,3.5)	3.16 ± 0.78	3.5(2.5,4)	3.28 ± 0.56	3.33(3,3.67)	3.08 ± 1.06	3(2,4)
Region	Northeast China	3.28 ± 0.46	3.33(3,3.67)	3.33 ± 0.69	3.5(3,4)	3.31 ± 0.54	3.33(3,3.67)	3.19 ± 1.01	4(3,4)
	North China	3.14 ± 0.55	3.22(2.89,3.56)	3.15 ± 0.81	3.5(2.5,4)	3.25 ± 0.56	3.33(3,3.67)	3.02 ± 1.06	3(2,4)
	East China	3.23 ± 0.5	3.33(2.94,3.61)	3.2 ± 0.74	3.5(2.5,4)	3.29 ± 0.55	3.33(3,3.67)	3.2 ± 0.99	4(3,4)
	South Central China	3.03 ± 0.65	3.17(2.67,3.56)	2.98 ± 0.85	3(2.5,3.5)	3.23 ± 0.59	3.33(3,3.67)	2.88 ± 1.13	3(2,4)
	Southwest China	3.02 ± 0.66	3.11(2.56,3.56)	2.97 ± 0.88	3(2.5,3.5)	3.21 ± 0.59	3.33(3,3.67)	2.87 ± 1.12	3(2,4)
	Northwest China	3.14 ± 0.58	3.22(2.83,3.56)	3.18 ± 0.79	3.5(3,4)	3.26 ± 0.56	3.33(3,3.67)	2.98 ± 1.07	3(2,4)
Grade	Grade 1	3.07 ± 0.63	3.22(2.67,3.56)	3.01 ± 0.85	3(2.5,3.5)	3.25 ± 0.58	3.33(3,3.67)	2.95 ± 1.12	3(2,4)
	Grade 2	3.15 ± 0.57	3.22(2.83,3.56)	3.13 ± 0.79	3.5(2.5,4)	3.25 ± 0.56	3.33(3,3.67)	3.05 ± 1.05	3(2,4)
	Grade 3	3.27 ± 0.46	3.33(3,3.61)	3.28 ± 0.71	3.5(3,4)	3.31 ± 0.55	3.33(3,3.67)	3.22 ± 0.97	4(3,4)
	Grade 4	3.2 ± 0.51	3.28(2.94,3.56)	3.18 ± 0.76	3.5(2.5,4)	3.26 ± 0.53	3.33(3,3.67)	3.17 ± 1.01	4(2,4)
	Grade 5	3.19 ± 0.57	3.28(2.89,3.61)	3.17 ± 0.79	3.5(3,4)	3.31 ± 0.62	3.33(3,3.67)	3.11 ± 1.01	3(2,4)
School	Project 985 University	2.68 ± 0.74	2.67(2.06,3.28)	2.6 ± 0.96	2.5(2,3.5)	3.09 ± 0.62	3(2.67,3.67)	2.35 ± 1.16	2(1,3)
	Project 211 University	3.21 ± 0.51	3.28(2.89,3.56)	3.21 ± 0.74	3.5(2.5,4)	3.27 ± 0.57	3.33(3,3.67)	3.14 ± 1.02	3(2,4)
	State University	3.28 ± 0.45	3.33(3,3.61)	3.26 ± 0.7	3.5(3,4)	3.3 ± 0.54	3.33(3,3.67)	3.27 ± 0.93	4(3,4)
	Private University	3.28 ± 0.43	3.33(3,3.61)	3.29 ± 0.66	3.5(3,4)	3.33 ± 0.52	3.33(3,3.67)	3.23 ± 0.97	4(3,4)
Duration of aerobic exercise per week	Less than 2 h	3.14 ± 0.59	3.22(2.83,3.56)	3.12 ± 0.8	3.5(2.5,4)	3.25 ± 0.58	3.33(3,3.67)	3.07 ± 1.06	3(2,4)
	2 ~ 4 h	3.21 ± 0.51	3.28(2.89,3.56)	3.19 ± 0.75	3.5(2.5,4)	3.31 ± 0.53	3.33(3,3.67)	3.13 ± 1.03	3(2,4)
	4 ~ 6 h	3.21 ± 0.51	3.33(2.89,3.56)	3.21 ± 0.76	3.5(3,4)	3.28 ± 0.54	3.33(3,3.67)	3.15 ± 1	3(2,4)
	More than 6 h	3.25 ± 0.49	3.33(2.94,3.61)	3.28 ± 0.73	3.5(3,4)	3.32 ± 0.55	3.33(3,3.67)	3.15 ± 1.01	3.5(2,4)
Duration of anaerobic exercise per week	Less than 1 h	2.95 ± 0.68	3.06(2.44,3.5)	2.91 ± 0.91	3(2,3.5)	3.17 ± 0.6	3.33(2.67,3.67)	2.77 ± 1.16	3(2,4)
	1 ~ 2 h	3.26 ± 0.47	3.33(2.94,3.61)	3.23 ± 0.71	3.5(3,4)	3.32 ± 0.53	3.33(3,3.67)	3.22 ± 0.96	4(3,4)
	2 ~ 3 h	3.28 ± 0.44	3.33(3,3.61)	3.29 ± 0.68	3.5(3,4)	3.31 ± 0.52	3.33(3,3.67)	3.23 ± 0.96	4(3,4)
	More than 3 h	3.28 ± 0.48	3.33(3,3.65)	3.26 ± 0.71	3.5(3,4)	3.31 ± 0.56	3.33(3,3.67)	3.26 ± 0.97	4(3,4)

Subsequent investigations into the factors influencing the overall score and the scores of the 3 components were conducted. The results of the univariate analysis (see Supplementary Table S5) indicated that region, grade, school, weekly aerobic exercise duration, and weekly anaerobic exercise duration were significant factors influencing the overall practice score (*P* < 0.001). These factors also affected the scores for PARI risk assessment (*P* < 0.001), while grade (*P* < 0.05), school, weekly aerobic exercise duration (*P* < 0.05), and weekly anaerobic exercise duration influenced the PARI preventive measures score. Region, grade, school, and weekly anaerobic exercise duration impacted the scores for PARI emergency response measures (*P* < 0.001). To mitigate the inter-correlation among independent variables, multivariate linear regression analysis was employed, which discounted the effect of grade on PARI preventive measures (see Supplementary Table S6).

**Figure 4:**
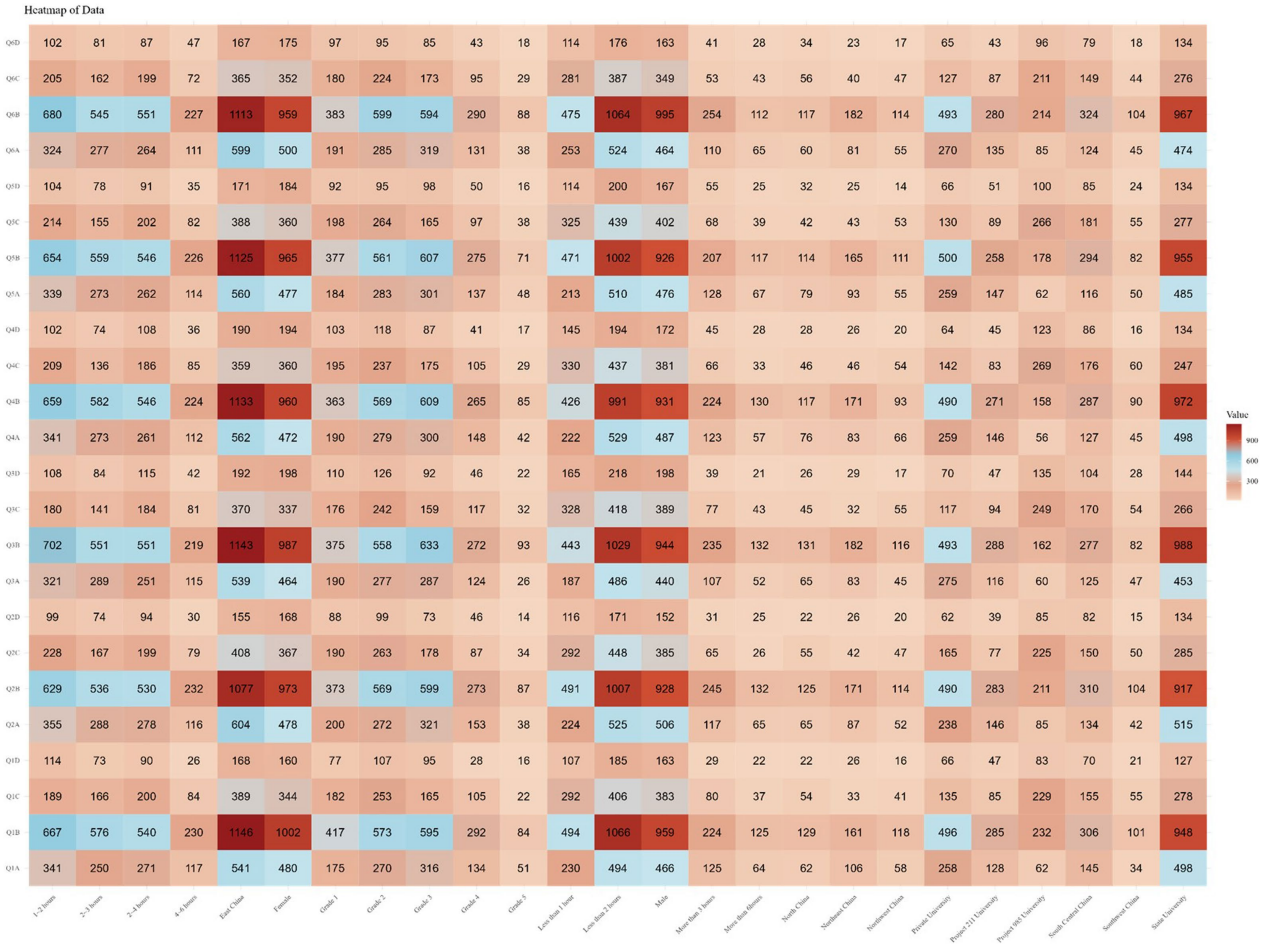
Number of people with each option for each question by subgroup.

Finally, focusing on each individual question, a majority of respondents demonstrated a high rate of good practice (selecting “always” or “often”), ranging between 70 and 80% for each question. The percentage of respondents selecting each option for every question is illustrated in Supplementary Figure S4. Subsequently, the selection frequencies for each option of every question in different subgroups were analyzed, revealing that the response patterns closely paralleled the overall trend, as shown in Figure 4.

### 3.5 Correlation among scores of knowledges, attitude and behavior

Integrating the scores from the knowledge, attitude, and practice sections, a Pearson correlation analysis was conducted. This revealed a positive correlation between overall scores and the three components, as well as between knowledge and attitude, accompanied by a negative correlation of both with practice (*P* < 0.001).

Subsequently, subgroup analyses were performed to evaluate the consistency of these correlations across different subgroups (see Supplementary Table S7). It was found that in the case of 5th-grade students, the majority of these correlations were not significant. Similarly, in the section pertaining to PARI preventive measures, most correlations were found to be insignificant. Additionally, for students engaging in ≥2 h of weekly aerobic exercise or ≥1 h of weekly anaerobic exercise, the majority of these correlations were also not significant.

### 3.6 Public health education demands of PARI prevention

**Figure 5:**
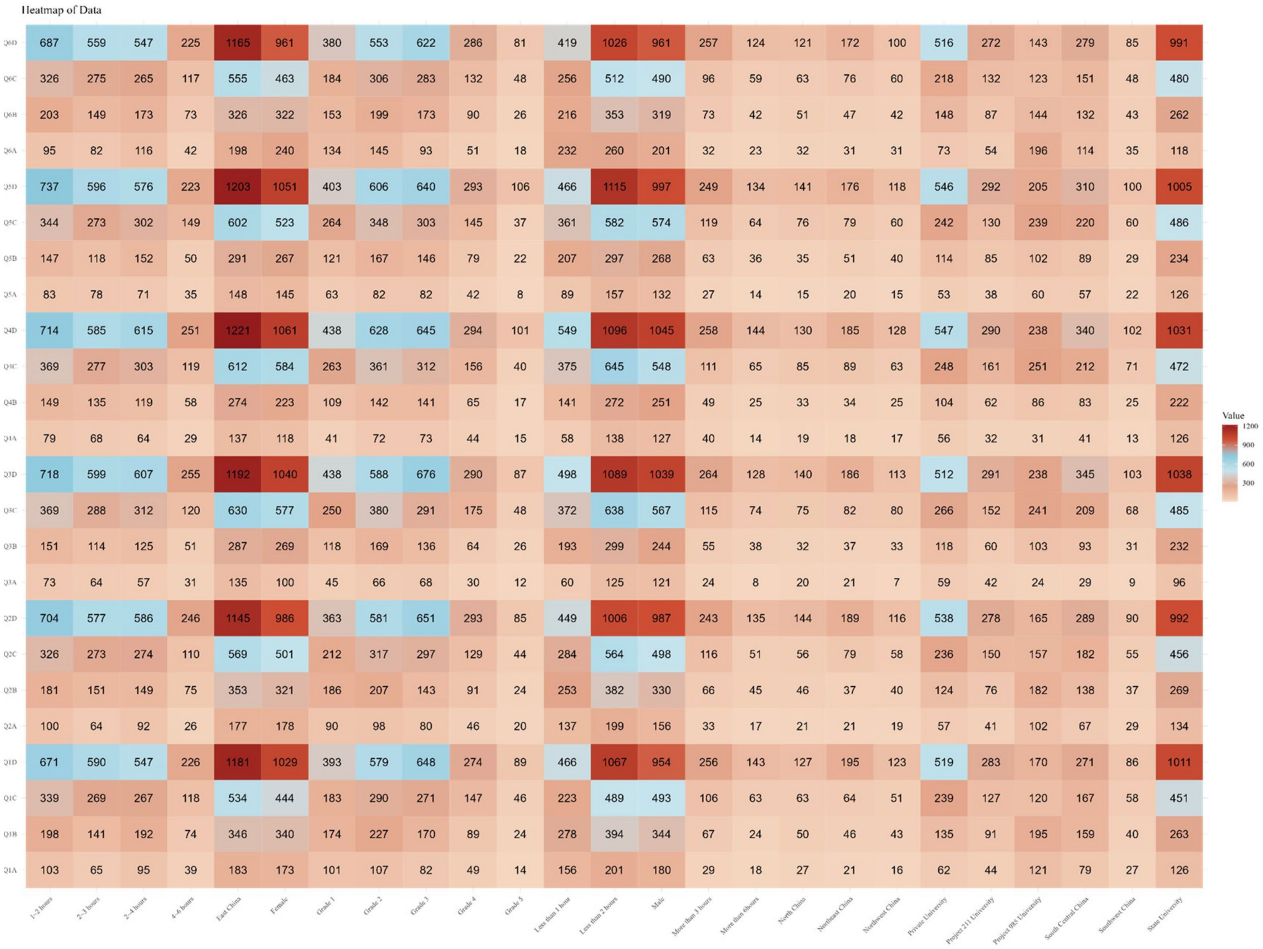
Needs for scientific popularization.

In the ranking question regarding preferred teaching methods, respondents predominantly favored “guiding students to independently discover, propose, and solve problems, thereby stimulating their learning interest and initiative.” Conversely, there was a general lack of preference for “organizing students into teams to collaboratively research acute sports injury prevention and management outside of class hours, culminating in the presentation of the team’s research findings” (see Figure 5A). The responses from various subgroups aligned with these findings (see Supplementary Table S8).

In the multiple-choice question about desired training content, respondents widely preferred training on “the application of the RICE principle” (56.86%), while only 33.26% expressed a preference for training on “indicators related to cardiovascular events during exercise” (see Figure 5B). The responses from various subgroups were consistent with this trend (see Supplementary Table S9).

In the multiple-choice question about the preferred formats for training delivery in the multiple-choice question, respondents commonly favored “relevant books and literature” (58.83%) and “informational brochures on relevant knowledge” (56.48%), whereas only 39.25% preferred the “lecture” format (see Figure 5C). The responses across different subgroups were in agreement with these preferences (see Supplementary Table S10).

In the multiple-choice question about preferred locations for seeing related promotional content, respondents most desired to see such content in “school campuses” (49.79%), while only 29.39% wished to see it in “public sports venues” (see Figure 5D). The responses from various subgroups corresponded with these findings (see Supplementary Table S11).

## 4 Discussion

Physical activity-related injuries (PARI) are increasingly prevalent among Chinese university students (12). These injuries encompass both acute damages, such as sprains and fractures, and chronic harms caused by repetitive stress (2). Not only do these injuries hinder daily activities, but they also may lead to long-term health issues (4). Despite the significant impact of these injuries, public knowledge about their prevention and management is generally limited, highlighting the need for enhanced public health education and more resources (1).

Our study found that while approximately half of the Chinese university students lack knowledge about PARI and hold relatively negative attitudes, they still demonstrate good behavioral practices. In terms of preventive measures, the Knowledge, Attitudes, and Practices (KAP) levels were relatively high, but the KAP levels for emergency response measures were lower. These results underscore the need to elevate public health education among students about PARI prevention and emergency response. In the public health educational needs survey, participants expressed a preference for “guiding students to independently discover, propose, and solve problems.” In terms of training content, students showed a preference for learning the RICE principles. Meanwhile, in terms of training formats, they favored “relevant books and literature” and “information booklets on relevant knowledge.” Furthermore, students preferred seeing related promotional content on “school campuses” rather than in “public sports venues.”

### 4.1 Knowledge of PARI prevention

In the knowledge aspect, participants’ average knowledge score was 1.84 ± 0.52, indicating a generally poor understanding of PARI among university students (20). This might reflect the limited coverage of PARI in standard public health education and physical education curricula, suggesting room for improvement in disseminating this knowledge among university students (21). The students’ understanding of PARI prevention measures was relatively higher, with an average score of 1.88 ± 0.67. This could be due to more emphasis in physical education and public health education on more intuitive and straightforward preventive measures like warm-up exercises and the use of safety equipment (20). In contrast, the average score for PARI emergency measures was lower at 1.81 ± 0.90, indicating a gap in knowledge. This gap may be due to the professional skills and knowledge required for emergency measures, such as first aid and emergency decision-making, which may not often be included in basic health and physical education (21).

Notably, respondents from ‘985 Project’ universities scored the highest (2.3 ± 0.7). This is attributed to these institutions’ higher educational resources and teaching quality, offering more comprehensive health and physical education programs. More importantly, these universities typically prioritize prevention in health and physical education curricula, recognizing the long-term benefits of preventing sports injuries. Compared to more complex emergency techniques, this emphasis makes it easier for students to grasp and retain prevention measures (22).

Multivariate linear regression analysis found that region, grade, school, and weekly anaerobic exercise time significantly influence the overall average score for PARI knowledge (*P* < 0.001). Regionally, differences in educational resources and sports facilities among regions may be one of the reasons for the discrepancy in PARI knowledge (23). Some areas, with more advanced sports infrastructure and diverse sports activities, provide better opportunities for education and practice in PARI prevention and management. Cultural and lifestyle differences among regions might also affect students’ attitudes toward sports safety (24, 25). Regarding grade, as students’ progress through university, their mastery of knowledge shows a V-shaped change. This might be because students are more attentive to PARI in their early university years, but this attention may wane over time if they do not experience related injuries directly. However, in higher grades, due to personal or surrounding sports injury experiences, students may re-acknowledge the importance of PARI and enhance their learning of related knowledge (26). Additionally, as students’ roles in sports activities and clubs change, their practical understanding and application of PARI might also strengthen, reflecting their dynamic attention to health and safety issues (26). Concerning anaerobic exercise time, the longer students participate in anaerobic activities like weightlifting and sprinting, the higher the risk of injury and their awareness of PARI. These activities necessitate more attention to injury prevention and response measures, thus influencing students’ knowledge level in this area (12).

### 4.2 Attitude of PARI prevention

The average attitude score was 2.12 ± 0.51. This score was fairly consistent across different aspects of PARI, including risk assessment (2.11 ± 0.66), preventive measures (2.14 ± 0.61), and emergency measures (2.10 ± 0.87). Notably, participants from ‘985 Project’ universities scored the highest (2.59 ± 0.69), indicating a more positive attitude toward these aspects of PARI. As 985 universities, they often provide research and practical opportunities for students to deepen their understanding of PARI and witness or experience its potential impacts firsthand, thereby reinforcing the importance of prevention and response measures.

The PARI prevention measures score for students from 985 universities was high at 2.73 ± 0.74, reflecting their positive attitude, thanks to the multifaceted educational and environmental measures taken by these institutions. Nine hundred eighty-five universities often have advanced sports facilities and resources, providing students with practical experiences and demonstrations of injury prevention techniques, enhancing their understanding and application of these measures. Furthermore, the faculty at ‘985 Project’ universities, often being leaders in their respective fields, can provide high-quality and up-to-date information on best practices for injury prevention. More importantly, the overall environment at these universities, characterized by a culture of health and safety awareness and positive peer influence, plays a crucial role in shaping students’ attitudes toward PARI prevention measures (27, 28).

Multivariate linear regression analysis revealed significant effects of factors such as region, academic year, institution, and the weekly duration of aerobic exercise on the cumulative scores (*P* < 0.001). These factors similarly influenced scores for PARI risk assessment (*P* < 0.001), PARI preventive strategies (*P* < 0.001), and PARI emergency procedures (*P* < 0.001).

Regionally, the impact of the area highlights the role of geographical differences in educational policies, availability of sports facilities, and cultural attitudes toward sports and health, suggesting that regions with more resources and greater emphasis on sports safety provide more comprehensive PARI education, leading to higher scores (29). Regarding academic year, we observed a V-shaped trend, which may partly be attributed to changes in social influences, environmental factors, and the impact of educational and promotional activities. Specifically, at the beginning of university, students might have a heightened attitude toward PARI due to the novelty of the campus environment and active health promotion. However, as they adapt to university life, this initial high level of concern may gradually diminish. Yet, in the senior years, facing long-term considerations for future careers and health, students might re-elevate their attention to PARI due to new educational activities or personal growth experiences. This reflects the dynamic change in university students’ attitudes toward health and safety issues throughout their academic journey and also highlights the significant role of educational and social environments in shaping their attitudes (30). Finally, the time spent on anaerobic exercise, known for its higher intensity and greater risk of injury, might heighten students’ awareness and understanding of PARI, especially in prevention and emergency practices.

### 4.3 Practice of PARI prevention

The practice part’s average score was 3.18 ± 0.55. Upon studying scores for different elements, it was found that scores for assessing PARI risks (3.16 ± 0.78), implementing PARI preventive actions (3.27 ± 0.56), and executing PARI emergency interventions (3.10 ± 1.04) were essentially similar.

However, we noted that respondents from ‘985 Project’ universities (2.68 ± 0.74) and those engaging in less than 1 h of anaerobic exercise per week (2.95 ± 0.68) had poorer performance in practice. For students from ‘985 Project’ universities, their lower scores in practice compared to the general trend could be attributed to the gap between theoretical knowledge and practical application. These prestigious institutions, while excelling in academics and research, might not place enough emphasis on practical training in physical activity-related injuries (PARI), leading to a gap between the knowledge students possess and how to apply this knowledge in real-world scenarios (31). Additionally, these students might be overconfident in their practical skills due to their strong theoretical knowledge, which may not effectively translate into practical efficacy (1).

For students engaging in less than 1 h of anaerobic exercise per week, their lower scores in practice could be due to limited exposure and experience in handling PARI. Moreover, these students might reduce their focus on preventive and emergency measures, perceiving a lower risk of injury due to less participation in sports activities (1). Therefore, limited opportunities to engage in sports or physical activities result in fewer chances to learn and apply practical PARI measures.

Multivariate linear regression analysis showed that the academic year, institution, weekly aerobic exercise time, and weekly anaerobic exercise time significantly influenced the overall practice score (*P* < 0.001). These variables also similarly affected the scores for assessing PARI risks (*P* < 0.001). Regional differences in health and sports infrastructure, as well as cultural attitudes toward safety, greatly affect PARI practices.

In terms of academic year, we noticed an inverted-V trend. This may be due to students exhibiting higher sensitivity and adaptability to new environments and health information upon first entering university, hence being more active and proactive in preventing PARI. They are likely to more diligently follow sports safety rules, participate in public health education activities, and take other preventive measures. However, as they adapt to university life, this initial vigilance may gradually diminish. Concurrently, with the progression in academic years, students might start neglecting the importance of preventing PARI due to overconfidence or underestimation of risks. This overconfidence might stem from an overestimation of their physical abilities and risk assessment, leading them to engage in riskier behaviors during sports and reduce the preventive measures previously taken (32).

However, regionally, geographical location does not influence the measures taken for PARI prevention. This indicates that there is little variation between regions in terms of preventive measures, suggesting that health and physical education curricula have adopted standardized methods, thus enabling a uniform understanding and practice regardless of geographical differences.

For the emergency intervention scores of PARI, region, academic year, institution, and weekly duration of aerobic exercise all had an impact (*P* < 0.001), except for the duration of aerobic exercise. The significance of anaerobic exercise is often linked with higher injury risks and more frequent emergency situations, highlighting its importance in developing emergency capabilities (33). In contrast, the duration of aerobic exercise, usually lower in intensity and associated with lesser injury risk, seems to have a lesser impact on emergency intervention skills, indicating that the type and intensity of physical exercise have distinctly different impacts on emergency response readiness.

### 4.4 Correlation among scores of knowledges, attitude and behavior

The Pearson correlation analysis combined with the study results indicates a nuanced relationship between knowledge, attitudes, and practices concerning Physical Activity-Related Injuries (PARI). While there is a positive correlation between knowledge and attitudes, indicating that increased awareness of PARI positively influences attitudes toward its prevention and management, there is a puzzling negative correlation between these factors and actual practices.

This disparity suggests a ‘knowledge-attitude-practice gap,’ where increased awareness and improved attitudes do not necessarily lead to corresponding behavioral changes. Reasons for this gap may be multifaceted, such as psychological barriers like fear of injury or lack of self-efficacy (34), perceived barriers to practice, or a complacency effect due to increased knowledge creating a false sense of security (35). Moreover, the practical application of PARI knowledge and attitudes is complex, often constrained by contextual and environmental factors like limited resources or insufficient practical training opportunities. Therefore, this correlation analysis emphasizes a key challenge in PARI management: transforming theoretical knowledge and positive attitudes into effective practical actions.

### 4.5 Public health education demands of PARI prevention

Upon completing the analysis of undergraduate students’ levels and influencing factors of knowledge, beliefs, and practices regarding PARI, we further investigated the views and needs of university students regarding the implementation of PARI training and promotion.

In the context of preferred training methods, participants demonstrated a preference for “guiding students to independently discover, pose, and solve problems,” indicating that autonomous and inquiry-based learning might be more effective in PARI prevention education (36). In contrast, the approach of “organizing students to collaboratively research the prevention and treatment of acute sports injuries outside of class” was less popular. This may relate to students’ time constraints and the feasibility of teamwork. Most university students juggle between heavy academic loads, part-time jobs, and personal life, making involvement in additional extracurricular projects potentially burdensome, especially when not directly related to their primary academic goals and career planning (15, 37). Moreover, effective teamwork requires good coordination and communication among members, which might be challenging among students with different academic backgrounds, schedules, and geographical locations.

In terms of desired training content, participants showed a stronger preference for training in the implementation of the RICE principles (56.86%) over training for cardiovascular incidents in physical activities (33.26%). This could be due to the practicality and immediate applicability of the RICE method. The RICE principle, a well-known direct approach for treating acute sports injuries, holds significant practicality and operability for individuals involved in physical activities. Conversely, interest in recognizing signs of cardiovascular events is lower, possibly because it’s perceived as lacking immediacy or direct relevance. Although identifying cardiovascular events is crucial, they may be less common or not directly linked to participants’ routine physical activities (38).

Regarding preferred training formats, the majority of participants favored “relevant books and publications” (58.83%) and “educational pamphlets on related topics” (56.48%), indicating a preference for self-learning and easily accessible learning resources. These formats offer flexibility in self-paced learning and the ability to consult materials as needed, providing convenient and enduring resources for learners (39). The lower preference for “workshops” (39.25%) may reflect practical constraints such as availability of time or scheduling conflicts. Workshops are usually scheduled at specific times, but with students’ already tight schedules, finding additional time for fixed-schedule workshops can be challenging. Moreover, workshops often require several hours or more, and for students with limited time resources, participation might mean sacrificing other important activities or study time, especially when the workshop content is not directly related to their main interests or academic requirements. Most importantly, compared to more flexible learning methods, such as online courses, workshops typically lack flexibility in scheduling, not allowing students to adjust freely according to their own timetables (40).

As for preferred locations for PARI promotion, the highest proportion of respondents favored encountering promotional materials “within educational institutions” (49.79%), highlighting the importance of educational institutions as primary sources of credible and relevant information. This preference underscores the role of educational institutions in health and safety promotion, reflecting the convenience and frequent contact these venues provide for learners (41). In contrast, interest in receiving such information at “public sports facilities” (29.39%) was lower, possibly due to university students spending less time at public sports facilities compared to campuses.

As with any survey-based research, some possible limitations should be noted in this study. Firstly, the sample may not be entirely representative of all Chinese undergraduate students, potentially limiting the generalizability of the results. Secondly, the reliance on self-reported data could introduce biases, as respondents may not accurately recall or report their knowledge, attitudes, and practices regarding PARI. Thirdly, the cross-sectional nature of the study restricts the ability to establish causality between the identified factors and PARI prevention practices. Additionally, the study might not have accounted for all possible confounding variables that could influence the outcomes, such as socio-economic status or prior exposure to public health education. Finally, the study’s focus on undergraduates means that the findings may not be applicable to other groups, such as professionals or amateur sports enthusiasts, who might have different levels of awareness and practice concerning PARI.

As with any survey-based research, this study should also acknowledge some potential limitations. Firstly, the reliance on self-reported data could introduce biases, as respondents may not accurately recall or report their knowledge, attitudes, and practices regarding PARI. Secondly, the cross-sectional nature of the study limits the ability to establish causality between identified factors and PARI prevention measures. Additionally, the focus on university students means that the findings may not be applicable to other groups, such as professionals or amateur sports enthusiasts, who might have different levels of awareness and practice concerning PARI.

There are also strengths to consider, such as the nationwide scope of the survey among university students, which provides broad coverage, and the fact that our research addresses issues highlighted in the latest guidelines, making the findings representative and typical. Lastly, undergraduates, as a large and accessible group, provide a unique sample that can be used to assess the effectiveness of health education programs. The research outcomes can guide broader public health strategies.

The theoretical implications of this study were that a high level of knowledge and good attitude do not always directly affect good practice. When establishing an educational model for PARI public health education, especially for undergraduates, practice-oriented courses can be offered, such as organizing field visits, participating in volunteer services or conducting practical projects to help college students translate what they have learned into practical actions.

In addition, the research results also highlight the impact of population and behavioral factors on healthy behavior. When establishing the education model, the course content and education methods can be adjusted according to local conditions. For example, PARI public health education courses implemented in different regions can be adjusted according to the specific local environment and needs, while for different types of universities, corresponding educational models can be designed according to their socio-economic background and the characteristics of student groups.

## 5 Conclusion

This study surveyed 3,957 Chinese undergraduates, uncovering a gap in knowledge, attitudes, and practices regarding PARI among this group. Despite showing proficiency in practical application, their understanding of PARI is not comprehensive, and their attitudes are neutral. The research highlights the urgent need for more practical and application-focused public health educational interventions and resources in university environments. By addressing these gaps and utilizing students’ preferred learning methods, public health educational institutions can strengthen PARI prevention and management, thereby enhancing the overall health and safety of university students.

Therefore, in the future, when building a teaching model of PARI’s public health education for college students, we need to pay more attention to the connection among knowledge, attitude and practice, and choose the guided teaching methods and content that undergraduates generally want to focus on and the way they prefer to learn.

## Data availability statement

The raw data supporting the conclusions of this article will be made available by the authors, without undue reservation.

## Ethics statement

The studies involving humans were approved by Ethics Committee on Third Xiangya Hospital of Central South University. The studies were conducted in accordance with the local legislation and institutional requirements. The participants provided their written informed consent to participate in this study.

## Author contributions

YK: Formal analysis, Funding acquisition, Validation, Visualization, Writing – original draft, Writing – review & editing. XZ: Writing – original draft. YY: Data curation, Formal analysis, Methodology, Writing – original draft. HX: Investigation, Methodology, Writing – original draft. LM: Investigation, Writing – original draft. YZ: Project administration, Supervision, Writing – review & editing.
