# Supplementary material for: Knowledge, attitudes, practice, and public health education demand regarding PARI prevention: a cross-sectional study among Chinese undergraduates
Source: Front Public Health. 2024 Jun 20;12:1387789. doi: 10.3389/fpubh.2024.1387789 (PMC11226332; doi:10.3389/fpubh.2024.1387789)
Supplement: Supplementary file 1 [file Data_Sheet_1.docx]

Figure S1 Distribution of IP Adress


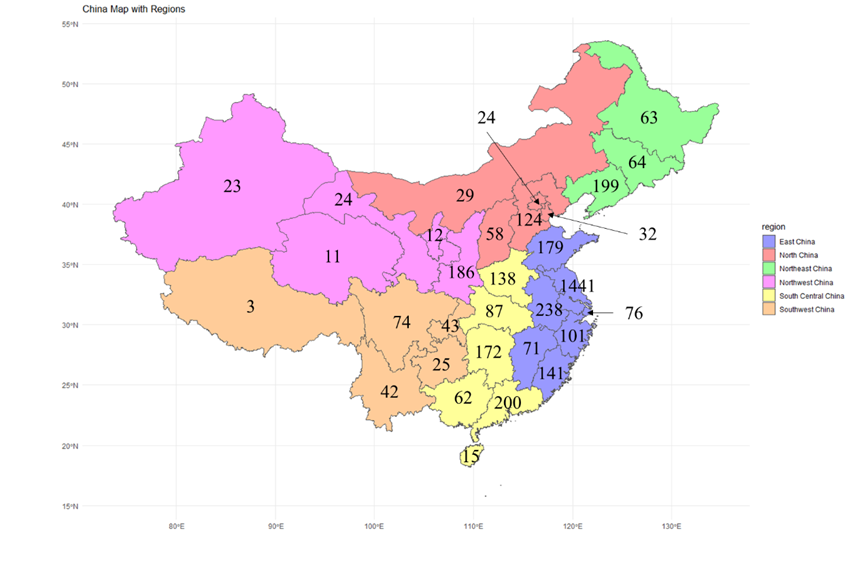


Figure S2 Percentage of People with Each Option for Each Question


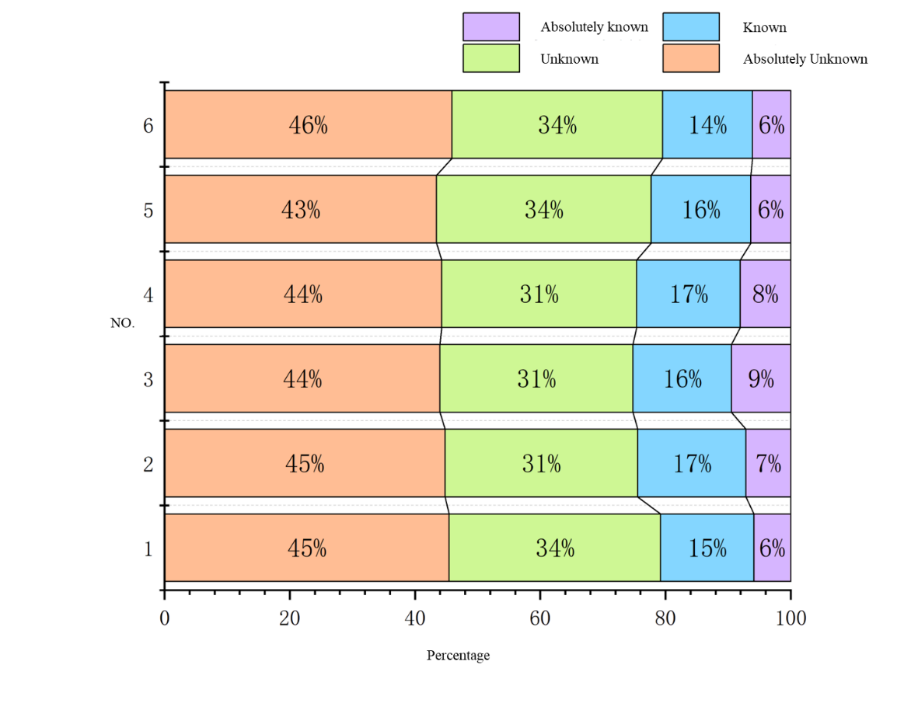


*Note:*

*1. The main indicators related to the risk of cardiovascular events during exercise are: (1) physical activity level; (2) conventional medical testing indicators: including heart rate, blood pressure, electrocardiogram, blood lipids, and blood glucose; and (3) recognizing the presence or absence of signs and symptoms of cardiovascular, metabolic, and renal diseases.*

*2. To minimize exercise-related risks, fitness testing should be performed*

*3. Warm-up or preparatory activities should be done before exercise, and finishing activities and stretching exercises should be done after exercise.*

*4. If you experience any discomfort such as chest pain, palpitations, or obvious pain in the joints and muscles during exercise, you should take corresponding measures immediately and seek medical attention if necessary.*

*5. Maintain a natural breathing state during resistance training, pay special attention to avoiding breath-holding, and provide appropriate protection when necessary.*

*6. Emergency treatment of sports injuries should follow the RICE principle, i.e. Rest, Ice, Compression and Elevation.*

Figure S3 Percentage of People with Each Option for Each Question


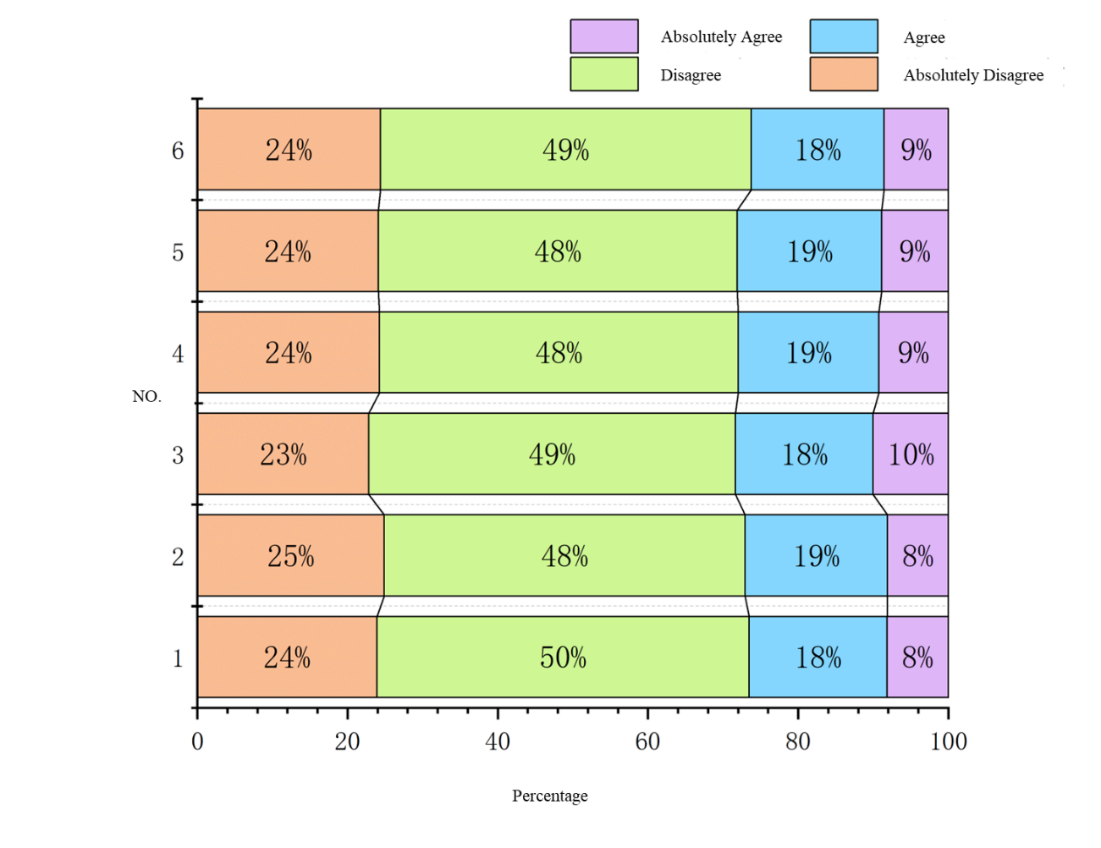


*Note:*

*1. I understand the indicators of risk of cardiovascular events during exercise and will exercise accordingly.*

*2. I will take the initiative to undergo physical fitness testing to minimize the associated risks.*

*3. I fully understand and will actively engage in warm-up or preparatory activities before exercise, as well as grooming activities and stretching exercises after exercise.*

*4. I am aware of symptoms that may occur during exercise and know that they may indicate an exercise problem and will immediately reduce the intensity of exercise or stop exercising.*

*5. I will take special care to maintain a natural breathing state during resistance training, avoid holding my breath and provide appropriate protection if needed.*

*6. I will follow the RICE principle of Rest, Ice, Compression, Elevation when responding to sports injuries.*

Figure S4 Percentage of People with Each Option for Each Question


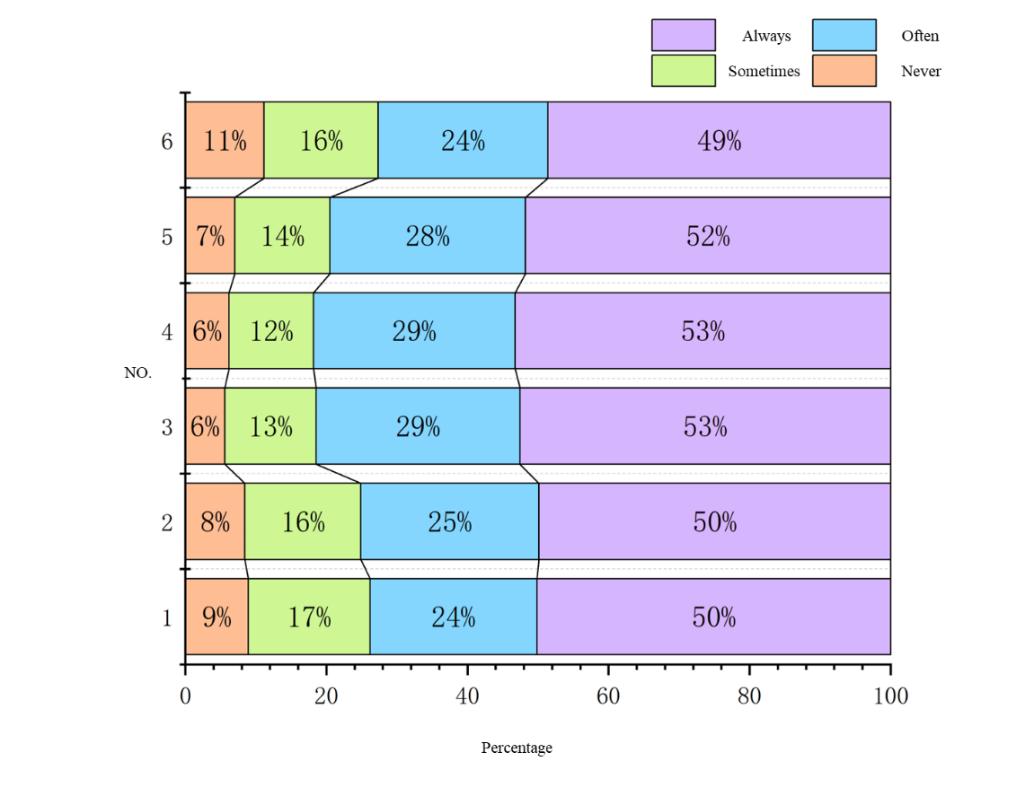


*Note:*

*1. When engaging in physical activity, I look for indicators to assess the risk of cardiovascular events during exercise.*

*2. When planning my exercise program, I will perform fitness tests to reduce exercise-related risks.*

*3. I do warm-up or preparatory activities before physical activity, and I do finishing activities and stretching exercises after physical activity.*

*4. I will reduce exercise intensity or stop exercise if I experience chest tightness or other discomfort during exercise.*

*5. I will keep breathing naturally and avoid holding my breath during resistance training.*

*6. I will skillfully use the RICE principle when someone is injured during exercise.*

Table S1 Results of ANOVA

| Variable | Total | PARI Risk Assessment | PARI Preventive Measures | PARI Emergency Measures |
| --- | --- | --- | --- | --- |
| Gender |  |  |  |  |
| Region | ●*** | ●*** | ●*** | ●* |
| Grade | ●*** | ●*** | ●*** |  |
| School | ●*** | ●*** | ●*** | ●*** |
| Duration of aerobic exercise per week |  |  |  |  |
| Duration of anaerobic exercise per week | ●*** | ●*** | ●*** | ●*** |

*Note: *** means P<0.001, **means P<0.01, *means P<0.05*

Table S2 Results of Multiple Linear Regression Analysis

| Variable | Total | | | | PARI Risk Assessment | | | | PARI Preventive Measures | | | | PARI Emergency Measures | | | |
| --- | --- | --- | --- | --- | --- | --- | --- | --- | --- | --- | --- | --- | --- | --- | --- | --- |
|  | β | SE | t | P | β | SE | t | P | β | SE | t | P | β | SE | t | P |
| Gender | 0.014 | 0.016 | 0.925 | 0.355 | 0.008 | 0.021 | 0.534 | 0.594 | 0.003 | 0.02 | 0.209 | 0.834 | 0.016 | 0.028 | 0.995 | 0.32 |
| Region | 0.049 | 0.007 | 3.24 | 0.001 | 0.035 | 0.01 | 2.28 | 0.023 | 0.041 | 0.009 | 2.776 | 0.006 | 0.027 | 0.013 | 1.713 | 0.087 |
| Grade | -0.043 | 0.007 | -2.876 | 0.004 | -0.017 | 0.01 | -1.118 | 0.264 | -0.048 | 0.009 | -3.245 | 0.001 | -0.026 | 0.013 | -1.664 | 0.096 |
| School | -0.288 | 0.008 | -18.77 | 0 | -0.206 | 0.011 | -13.02 | 0 | -0.305 | 0.01 | -20.14 | 0 | -0.116 | 0.015 | -7.211 | 0 |
| Duration of aerobic exercise per week | -0.005 | 0.009 | -0.33 | 0.741 | -0.006 | 0.012 | -0.416 | 0.677 | -0.018 | 0.011 | -1.195 | 0.232 | 0.01 | 0.016 | 0.611 | 0.541 |
| Duration of anaerobic exercise per week | -0.1 | 0.008 | -6.58 | 0 | -0.055 | 0.011 | -3.471 | 0.001 | -0.125 | 0.01 | -8.269 | 0 | -0.04 | 0.015 | -2.5 | 0.012 |

Table S3 Results of ANOVA

| Variable | Total | PARI Risk Assessment | PARI Preventive Measures | PARI Emergency Measures |
| --- | --- | --- | --- | --- |
| Gender |  |  |  |  |
| Region | ●*** | ●*** | ●*** | ●*** |
| Grade | ●*** | ●*** | ●*** | ●*** |
| School | ●*** | ●*** | ●*** | ●*** |
| Duration of aerobic exercise per week |  |  |  |  |
| Duration of anaerobic exercise per week | ●*** | ●*** | ●*** | ●*** |

*Note: *** means P<0.001, **means P<0.01, *means P<0.05*

Table S4 Results of Multiple Linear Regression Analysis

| Variable | Total | | | | PARI Risk Assessment | | | | PARI Preventive Measures | | | | PARI Emergency Measures | | | |
| --- | --- | --- | --- | --- | --- | --- | --- | --- | --- | --- | --- | --- | --- | --- | --- | --- |
|  | β | SE | t | P | β | SE | t | P | β | SE | t | P | β | SE | t | P |
| Gender | -0.011 | 0.015 | -0.72 | 0.472 | -0.008 | 0.02 | -0.535 | 0.593 | -0.012 | 0.018 | -0.82 | 0.412 | -0.004 | 0.027 | -0.274 | 0.784 |
| Region | 0.063 | 0.007 | 4.225 | 0 | 0.053 | 0.009 | 3.42 | 0.001 | 0.067 | 0.008 | 4.49 | 0 | 0.025 | 0.012 | 1.583 | 0.113 |
| Grade | -0.066 | 0.007 | -4.416 | 0 | -0.068 | 0.009 | -4.421 | 0 | -0.046 | 0.008 | -3.079 | 0.002 | -0.033 | 0.012 | -2.105 | 0.035 |
| School | -0.305 | 0.008 | -20.06 | 0 | -0.194 | 0.011 | -12.31 | 0 | -0.319 | 0.009 | -21.15 | 0 | -0.169 | 0.014 | -10.59 | 0 |
| Duration of aerobic exercise per week | -0.002 | 0.008 | -0.156 | 0.876 | -0.027 | 0.011 | -1.737 | 0.082 | -0.007 | 0.01 | -0.483 | 0.629 | 0.021 | 0.015 | 1.367 | 0.172 |
| Duration of anaerobic exercise per week | -0.101 | 0.008 | -6.682 | 0 | -0.079 | 0.011 | -5.058 | 0 | -0.11 | 0.009 | -7.351 | 0 | -0.041 | 0.014 | -2.606 | 0.009 |

Table S5 Results of ANOVA

| Variable | Total | PARI Risk Assessment | PARI Preventive Measures | PARI Emergency Measures |
| --- | --- | --- | --- | --- |
| Gender |  |  |  |  |
| Region | ●*** | ●*** |  | ●*** |
| Grade | ●*** | ●*** | ●* | ●*** |
| School | ●*** | ●*** | ●*** | ●*** |
| Duration of aerobic exercise per week | ●*** | ●*** | ●* |  |
| Duration of anaerobic exercise per week | ●*** | ●*** | ●*** | ●*** |

*Note: *** means P<0.001, **means P<0.01, *means P<0.05*

Table S6 Results of Multiple Linear Regression Analysis

| Variable | Total | | | | PARI Risk Assessment | | | | PARI Preventive Measures | | | | PARI Emergency Measures | | | |
| --- | --- | --- | --- | --- | --- | --- | --- | --- | --- | --- | --- | --- | --- | --- | --- | --- |
|  | β | SE | t | P | β | SE | t | P | β | SE | t | P | β | SE | t | P |
| Gender | 0.003 | 0.016 | 0.22 | 0.826 | 0.015 | 0.024 | 0.973 | 0.331 | 0.018 | 0.018 | 1.17 | 0.242 | -0.016 | 0.032 | -1.029 | 0.304 |
| Region | -0.065 | 0.007 | -4.354 | 0 | -0.055 | 0.011 | -3.577 | 0 | -0.018 | 0.008 | -1.108 | 0.268 | -0.052 | 0.014 | -3.422 | 0.001 |
| Grade | 0.069 | 0.007 | 4.644 | 0 | 0.063 | 0.011 | 4.132 | 0 | 0.017 | 0.008 | 1.077 | 0.281 | 0.053 | 0.014 | 3.482 | 0.001 |
| School | 0.284 | 0.009 | 18.78 | 0 | 0.226 | 0.012 | 14.568 | 0 | 0.111 | 0.009 | 6.926 | 0 | 0.221 | 0.017 | 14.207 | 0 |
| Duration of aerobic exercise per week | 0.038 | 0.009 | 2.571 | 0.01 | 0.045 | 0.013 | 2.961 | 0.003 | 0.031 | 0.01 | 1.957 | 0.05 | 0.01 | 0.018 | 0.663 | 0.507 |
| Duration of anaerobic exercise per week | 0.153 | 0.008 | 10.209 | 0 | 0.122 | 0.012 | 7.875 | 0 | 0.066 | 0.009 | 4.118 | 0 | 0.117 | 0.016 | 7.563 | 0 |

Table S7 Results of Pearson Correlation Analysis by Subgroup

| Subgroup | Total | | | | | | PARI Risk Assessment | | | | | | |
| --- | --- | --- | --- | --- | --- | --- | --- | --- | --- | --- | --- | --- | --- |
|  | KA | | KP | | AP | | KA | | KP | | AP | | |
|  | ρ | P | ρ | P | ρ | P | ρ | P | ρ | P | ρ | P |  |
| Overall | 0.457 | ＜0.001 | -0.257 | ＜0.001 | -0.297 | ＜0.001 | 0.226 | < 0.001 | -0.168 | < 0.001 | -0.194 | < 0.001 |  |
| Male | 0.448 | < 0.001 | -0.285 | < 0.001 | -0.322 | < 0.001 | 0.219 | < 0.001 | -0.16 | < 0.001 | -0.169 | < 0.001 |  |
| Female | 0.446 | < 0.001 | -0.273 | < 0.001 | -0.327 | < 0.001 | 0.233 | < 0.001 | -0.176 | < 0.001 | -0.218 | < 0.001 |  |
| Northeast China | 0.205 | < 0.001 | -0.115 | 0.038 | -0.146 | 0.008 | 0.126 | 0.023 | -0.031 | 0.571 | -0.114 | 0.039 |  |
| North China | 0.533 | < 0.001 | -0.339 | < 0.001 | -0.426 | < 0.001 | 0.282 | < 0.001 | -0.197 | 0.001 | -0.241 | < 0.001 |  |
| East China | 0.345 | < 0.001 | -0.218 | < 0.001 | -0.257 | < 0.001 | 0.158 | < 0.001 | -0.122 | < 0.001 | -0.14 | < 0.001 |  |
| South Central China | 0.606 | < 0.001 | -0.332 | < 0.001 | -0.367 | < 0.001 | 0.36 | < 0.001 | -0.255 | < 0.001 | -0.254 | < 0.001 |  |
| Southwest China | 0.481 | < 0.001 | -0.345 | < 0.001 | -0.455 | < 0.001 | 0.25 | < 0.001 | -0.249 | < 0.001 | -0.357 | < 0.001 |  |
| Northwest China | 0.502 | < 0.001 | -0.339 | < 0.001 | -0.26 | < 0.001 | 0.29 | < 0.001 | -0.188 | 0.004 | -0.114 | 0.083 |  |
| Grade 1 | 0.542 | < 0.001 | -0.337 | < 0.001 | -0.43 | < 0.001 | 0.336 | < 0.001 | -0.187 | < 0.001 | -0.307 | < 0.001 |  |
| Grade 2 | 0.494 | < 0.001 | -0.333 | < 0.001 | -0.333 | < 0.001 | 0.263 | < 0.001 | -0.221 | < 0.001 | -0.183 | < 0.001 |  |
| Grade 3 | 0.227 | < 0.001 | -0.151 | < 0.001 | -0.16 | < 0.001 | 0.085 | 0.004 | -0.106 | < 0.001 | -0.099 | < 0.001 |  |
| Grade 4 | 0.388 | < 0.001 | -0.266 | < 0.001 | -0.301 | < 0.001 | 0.17 | < 0.001 | -0.128 | 0.002 | -0.137 | 0.001 |  |
| Grade 5 | 0.551 | < 0.001 | 0.021 | 0.786 | -0.139 | 0.069 | 0.244 | 0.001 | -0.015 | 0.847 | -0.099 | 0.193 |  |
| Project 985 University | 0.66 | < 0.001 | -0.224 | < 0.001 | -0.298 | < 0.001 | 0.47 | < 0.001 | -0.173 | < 0.001 | -0.215 | < 0.001 |  |
| Project 211 University | 0.375 | < 0.001 | -0.331 | < 0.001 | -0.283 | < 0.001 | 0.158 | < 0.001 | -0.159 | < 0.001 | -0.194 | < 0.001 |  |
| State University | 0.179 | < 0.001 | -0.092 | < 0.001 | -0.136 | < 0.001 | 0.069 | 0.003 | -0.046 | 0.047 | -0.066 | 0.005 |  |
| Private University | 0.126 | < 0.001 | -0.043 | 0.18 | -0.098 | 0.002 | 0.072 | 0.025 | -0.074 | 0.023 | -0.076 | 0.019 |  |
| Less than 2 hours | 0.434 | < 0.001 | -0.326 | < 0.001 | -0.375 | < 0.001 | 0.211 | < 0.001 | -0.194 | < 0.001 | -0.241 | < 0.001 |  |
| 2~4 hours | 0.472 | < 0.001 | -0.247 | < 0.001 | -0.292 | < 0.001 | 0.25 | < 0.001 | -0.13 | < 0.001 | -0.16 | < 0.001 |  |
| 4~6 hours | 0.412 | < 0.001 | -0.142 | 0.002 | -0.202 | < 0.001 | 0.184 | < 0.001 | -0.135 | 0.004 | -0.091 | 0.051 |  |
| More than 6hours | 0.501 | < 0.001 | -0.187 | 0.003 | -0.162 | 0.011 | 0.318 | < 0.001 | -0.139 | 0.028 | -0.047 | 0.464 |  |
| Less than 1 hour | 0.614 | < 0.001 | -0.379 | < 0.001 | -0.429 | < 0.001 | 0.395 | < 0.001 | -0.291 | < 0.001 | -0.292 | < 0.001 |  |
| 1~2 hours | 0.292 | < 0.001 | -0.152 | < 0.001 | -0.189 | < 0.001 | 0.102 | < 0.001 | -0.074 | 0.008 | -0.112 | < 0.001 |  |
| 2~3 hours | 0.162 | < 0.001 | -0.07 | 0.023 | -0.137 | < 0.001 | 0.085 | 0.005 | -0.041 | 0.182 | -0.08 | 0.009 |  |
| More than 3 hours | 0.433 | < 0.001 | -0.17 | < 0.001 | -0.162 | < 0.001 | 0.224 | < 0.001 | -0.089 | 0.057 | -0.091 | 0.052 |  |
| Subgroup | PARI Preventive Measures | | | | | | PARI Emergency Measures | | | | | | |
|  | KA | | KP | | AP | | KA | | KP | | AP | | |
|  | ρ | P | ρ | P | ρ | P | ρ | P | ρ | P | ρ | P |  |
| Overall | 0.465 | < 0.001 | -0.051 | 0.001 | -0.077 | < 0.001 | 0.122 | < 0.001 | -0.077 | < 0.001 | -0.129 | < 0.001 |  |
| Male | 0.45 | < 0.001 | -0.059 | 0.009 | -0.09 | < 0.001 | 0.124 | < 0.001 | -0.096 | < 0.001 | -0.133 | < 0.001 |  |
| Female | 0.48 | < 0.001 | -0.043 | 0.054 | -0.064 | 0.004 | 0.121 | < 0.001 | -0.06 | 0.008 | -0.125 | < 0.001 |  |
| Northeast China | 0.157 | 0.004 | 0.017 | 0.759 | 0.006 | 0.921 | 0.022 | 0.689 | 0.069 | 0.216 | -0.041 | 0.457 |  |
| North China | 0.544 | < 0.001 | -0.045 | 0.463 | -0.077 | 0.212 | 0.193 | 0.002 | -0.105 | 0.086 | -0.144 | 0.018 |  |
| East China | 0.365 | < 0.001 | -0.016 | 0.455 | -0.068 | 0.001 | 0.084 | < 0.001 | -0.072 | < 0.001 | -0.104 | < 0.001 |  |
| South Central China | 0.652 | < 0.001 | -0.118 | 0.002 | -0.099 | 0.01 | 0.185 | < 0.001 | -0.094 | 0.015 | -0.16 | < 0.001 |  |
| Southwest China | 0.506 | < 0.001 | -0.015 | 0.833 | -0.134 | 0.052 | 0.266 | < 0.001 | -0.095 | 0.17 | -0.204 | 0.003 |  |
| Northwest China | 0.484 | < 0.001 | -0.134 | 0.04 | -0.01 | 0.882 | 0.16 | 0.015 | -0.14 | 0.033 | -0.079 | 0.232 |  |
| Grade 1 | 0.588 | < 0.001 | -0.056 | 0.104 | -0.147 | < 0.001 | 0.143 | < 0.001 | -0.129 | < 0.001 | -0.189 | < 0.001 |  |
| Grade 2 | 0.499 | < 0.001 | -0.101 | < 0.001 | -0.054 | 0.063 | 0.182 | < 0.001 | -0.072 | 0.013 | -0.16 | < 0.001 |  |
| Grade 3 | 0.474 | < 0.001 | 0.163 | 0.032 | -0.037 | 0.627 | 0.024 | 0.413 | -0.054 | 0.064 | -0.035 | 0.234 |  |
| Grade 4 | 0.251 | < 0.001 | 0.002 | 0.952 | -0.052 | 0.075 | 0.109 | 0.01 | -0.044 | 0.299 | -0.126 | 0.003 |  |
| Grade 5 | 0.39 | < 0.001 | -0.069 | 0.101 | -0.03 | 0.48 | 0.218 | 0.004 | -0.005 | 0.945 | -0.013 | 0.868 |  |
| Project 985 University | 0.692 | < 0.001 | 0.008 | 0.842 | 0 | 0.992 | 0.387 | < 0.001 | -0.012 | 0.771 | -0.136 | < 0.001 |  |
| Project 211 University | 0.426 | < 0.001 | -0.051 | 0.236 | -0.052 | 0.223 | 0.09 | 0.035 | -0.099 | 0.02 | -0.06 | 0.159 |  |
| State University | 0.187 | < 0.001 | 0.024 | 0.304 | -0.021 | 0.376 | 0.02 | 0.38 | -0.033 | 0.155 | -0.054 | 0.021 |  |
| Private University | 0.141 | < 0.001 | 0.01 | 0.761 | -0.021 | 0.514 | 0.019 | 0.552 | -0.007 | 0.823 | -0.052 | 0.109 |  |
| Less than 2 hours | 0.472 | < 0.001 | -0.098 | < 0.001 | -0.119 | < 0.001 | 0.126 | < 0.001 | -0.103 | < 0.001 | -0.148 | < 0.001 |  |
| 2~4 hours | 0.491 | < 0.001 | 0.009 | 0.759 | -0.03 | 0.318 | 0.161 | < 0.001 | -0.054 | 0.071 | -0.1 | < 0.001 |  |
| 4~6 hours | 0.338 | < 0.001 | 0.027 | 0.562 | 0 | 0.992 | 0.084 | 0.073 | 0.018 | 0.705 | -0.145 | 0.002 |  |
| More than 6hours | 0.494 | < 0.001 | 0.018 | 0.779 | -0.003 | 0.957 | 0.008 | 0.905 | -0.133 | 0.036 | -0.064 | 0.315 |  |
| Less than 1 hour | 0.654 | < 0.001 | -0.084 | 0.005 | -0.123 | < 0.001 | 0.257 | < 0.001 | -0.135 | < 0.001 | -0.232 | < 0.001 |  |
| 1~2 hours | 0.317 | < 0.001 | -0.006 | 0.841 | -0.031 | 0.266 | 0.044 | 0.114 | -0.028 | 0.312 | -0.065 | 0.018 |  |
| 2~3 hours | 0.156 | < 0.001 | 0.017 | 0.574 | 0.005 | 0.88 | 0.054 | 0.08 | -0.035 | 0.253 | -0.023 | 0.453 |  |
| More than 3 hours | 0.381 | < 0.001 | 0.056 | 0.228 | 0.017 | 0.716 | 0.088 | 0.059 | -0.023 | 0.622 | -0.085 | 0.07 |  |

*Note: KA means correlation between knowledge and attitude; AP means correlation between attitude and practice; KP means correlation between knowledge and practice*

Table S8 Sorting Results

| Subgroup | 1 | | 2 | | 3 | | 4 | |
| --- | --- | --- | --- | --- | --- | --- | --- | --- |
| Male | 2.62 ± 1.41 | 2(1,4) | 2.89 ± 1.8 | 2(1,5) | 2.98 ± 1.35 | 3(2,4) | 2.06 ± 1.22 | 2(1,3) |
| Female | 2.79 ± 1.45 | 3(2,4) | 2.86 ± 1.82 | 2(1,5) | 2.99 ± 1.37 | 3(2,4) | 1.97 ± 1.12 | 2(1,3) |
| Northeast China | 2.81 ± 1.31 | 3(2,4) | 2.99 ± 1.84 | 2(1,5) | 2.76 ± 1.17 | 3(2,4) | 1.87 ± 0.98 | 2(1,3) |
| North China | 2.76 ± 1.47 | 3(2,4) | 2.8 ± 1.72 | 2(2,4) | 3.15 ± 1.54 | 3(2,4) | 1.93 ± 1.14 | 2(1,3) |
| East China | 2.68 ± 1.32 | 3(1,4) | 2.95 ± 1.86 | 2(1,5) | 2.86 ± 1.23 | 3(2,3) | 1.93 ± 1.1 | 2(1,3) |
| South Central China | 2.78 ± 1.69 | 3(1,4) | 2.77 ± 1.75 | 2(1,4) | 3.3 ± 1.61 | 3(2,4) | 2.27 ± 1.36 | 2(1,3) |
| Southwest China | 2.5 ± 1.58 | 2(1,4) | 2.54 ± 1.62 | 2(1,3) | 3.21 ± 1.45 | 3(2,4) | 2.25 ± 1.22 | 2(1,3) |
| Northwest China | 2.6 ± 1.42 | 2(1,4) | 2.83 ± 1.7 | 2(2,4) | 3.07 ± 1.43 | 3(2,4) | 2.06 ± 1.23 | 2(1,3) |
| Grade 1 | 2.7 ± 1.56 | 3(1,4) | 2.87 ± 1.78 | 2(1,4) | 3.12 ± 1.47 | 3(2,4) | 2.14 ± 1.31 | 2(1,3) |
| Grade 2 | 2.75 ± 1.44 | 3(2,4) | 2.79 ± 1.76 | 2(1,4) | 3.03 ± 1.42 | 3(2,4) | 2.01 ± 1.18 | 2(1,3) |
| Grade 3 | 2.66 ± 1.26 | 3(2,4) | 2.9 ± 1.86 | 2(1,5) | 2.85 ± 1.18 | 3(2,4) | 1.93 ± 1.07 | 2(1,3) |
| Grade 4 | 2.78 ± 1.48 | 3(1,4) | 3.03 ± 1.88 | 2(1,5) | 2.9 ± 1.33 | 3(2,3.25) | 1.88 ± 1.03 | 2(1,2) |
| Grade 5 | 2.41 ± 1.49 | 2(1,4) | 2.89 ± 1.76 | 2(1.75,5) | 3.17 ± 1.46 | 3(2,4) | 2.23 ± 1.22 | 2(1,3) |
| Project 985 University | 2.83 ± 1.91 | 2(1,4) | 2.62 ± 1.6 | 2(1,4) | 3.94 ± 1.81 | 3(3,6) | 2.62 ± 1.49 | 2(1,4) |
| Project 211 University | 2.56 ± 1.37 | 2(1,4) | 2.75 ± 1.74 | 2(1,4) | 2.81 ± 1.32 | 3(2,4) | 2.04 ± 1.17 | 2(1,3) |
| State University | 2.7 ± 1.22 | 3(2,4) | 3.07 ± 1.91 | 2(1,5) | 2.77 ± 1.07 | 3(2,3) | 1.82 ± 1 | 1.5(1,2) |
| Private University | 2.62 ± 1.17 | 2(2,4) | 2.84 ± 1.79 | 2(1,5) | 2.71 ± 1.07 | 3(2,3) | 1.89 ± 1.03 | 2(1,3) |
| Less than 2 hours | 2.68 ± 1.46 | 3(1,4) | 2.83 ± 1.77 | 2(1,4) | 3 ± 1.41 | 3(2,4) | 2.05 ± 1.2 | 2(1,3) |
| 2~4 hours | 2.74 ± 1.41 | 3(1,4) | 2.89 ± 1.83 | 2(1,5) | 2.98 ± 1.29 | 3(2,4) | 1.99 ± 1.16 | 2(1,3) |
| 4~6 hours | 2.6 ± 1.33 | 2(1,4) | 2.86 ± 1.86 | 2(1,5) | 3 ± 1.37 | 3(2,4) | 1.96 ± 1.07 | 2(1,3) |
| More than 6hours | 2.92 ± 1.45 | 3(2,4) | 3.23 ± 1.94 | 2(2,5) | 2.88 ± 1.25 | 3(2,3) | 1.85 ± 1.06 | 1(1,3) |
| Less than 1 hour | 2.74 ± 1.69 | 2(1,4) | 2.71 ± 1.67 | 2(2,4) | 3.38 ± 1.65 | 3(2,4) | 2.32 ± 1.4 | 2(1,3) |
| 1~2 hours | 2.72 ± 1.29 | 3(2,4) | 3.02 ± 1.88 | 2(1,5) | 2.82 ± 1.19 | 3(2,3) | 1.9 ± 1.04 | 2(1,3) |
| 2~3 hours | 2.62 ± 1.19 | 3(2,4) | 2.85 ± 1.82 | 2(1,5) | 2.75 ± 1.07 | 3(2,3) | 1.81 ± 0.98 | 1(1,2) |
| More than 3 hours | 2.69 ± 1.37 | 3(1,4) | 3.04 ± 1.95 | 2(1,5) | 2.88 ± 1.29 | 3(2,4) | 1.95 ± 1.07 | 2(1,3) |
| Subgroup | 5 | | 6 | | 7 | |  | |
| Male | 2.63 ± 1.22 | 2(2,3) | 3.64 ± 2.28 | 3(1,6) | 3.78 ± 1.84 | 4(2,5) |  |  |
| Female | 2.58 ± 1.15 | 2(2,3) | 3.64 ± 2.26 | 3(1,6) | 4.01 ± 1.82 | 4(3,5) |  |  |
| Northeast China | 2.34 ± 0.96 | 2(2,3) | 3.21 ± 2.25 | 3(1,5) | 3.21 ± 1.67 | 3(2,5) |  |  |
| North China | 2.76 ± 1.26 | 2(2,3) | 3.46 ± 2.31 | 3(1,6) | 3.98 ± 1.82 | 4(2.25,5) |  |  |
| East China | 2.48 ± 1.06 | 2(2,3) | 3.59 ± 2.27 | 3(1,6) | 3.7 ± 1.73 | 4(2,5) |  |  |
| South Central China | 2.96 ± 1.44 | 2(2,4) | 4.08 ± 2.2 | 4(2,6) | 4.4 ± 1.94 | 5(3,6) |  |  |
| Southwest China | 2.83 ± 1.24 | 2(2,4) | 3.51 ± 2.34 | 3(1,6) | 4.26 ± 2.05 | 4(3,6) |  |  |
| Northwest China | 2.66 ± 1.17 | 2(2,3) | 3.74 ± 2.22 | 4(1,6) | 4.1 ± 1.85 | 4(3,5) |  |  |
| Grade 1 | 2.81 ± 1.29 | 2(2,3) | 4.02 ± 2.2 | 4(2,6) | 4.37 ± 1.92 | 5(3,6) |  |  |
| Grade 2 | 2.64 ± 1.23 | 2(2,3) | 3.56 ± 2.3 | 3(1,6) | 3.96 ± 1.84 | 4(3,5) |  |  |
| Grade 3 | 2.42 ± 0.95 | 2(2,3) | 3.46 ± 2.27 | 3(1,6) | 3.44 ± 1.68 | 4(2,5) |  |  |
| Grade 4 | 2.55 ± 1.18 | 2(2,3) | 3.69 ± 2.27 | 4(1,6) | 3.79 ± 1.72 | 4(2,5) |  |  |
| Grade 5 | 2.72 ± 1.36 | 2(2,3) | 3.36 ± 2.2 | 3(1,5.5) | 3.84 ± 1.88 | 4(2,5) |  |  |
| Project 985 University | 3.59 ± 1.63 | 3(2,5) | 4.56 ± 2.03 | 5(3,6) | 5.01 ± 1.84 | 5(4,7) |  |  |
| Project 211 University | 2.55 ± 1.11 | 2(2,3) | 3.42 ± 2.23 | 3(1,6) | 3.6 ± 1.8 | 4(2,5) |  |  |
| State University | 2.36 ± 0.93 | 2(2,3) | 3.53 ± 2.3 | 3(1,6) | 3.51 ± 1.64 | 4(2,5) |  |  |
| Private University | 2.35 ± 0.78 | 2(2,3) | 3.22 ± 2.22 | 3(1,5) | 3.31 ± 1.55 | 3(2,5) |  |  |
| Less than 2 hours | 2.67 ± 1.25 | 2(2,3) | 3.59 ± 2.25 | 3(1,6) | 4.01 ± 1.85 | 4(3,5) |  |  |
| 2~4 hours | 2.55 ± 1.12 | 2(2,3) | 3.67 ± 2.29 | 4(1,6) | 3.87 ± 1.83 | 4(2,5) |  |  |
| 4~6 hours | 2.47 ± 1.07 | 2(2,3) | 3.69 ± 2.29 | 3(1,6) | 3.49 ± 1.81 | 3(2,5) |  |  |
| More than 6hours | 2.53 ± 1.05 | 2(2,3) | 3.9 ± 2.33 | 4(1,6) | 3.76 ± 1.66 | 4(3,5) |  |  |
| Less than 1 hour | 3.08 ± 1.5 | 3(2,4) | 4.01 ± 2.2 | 4(2,6) | 4.56 ± 1.93 | 5(3,6) |  |  |
| 1~2 hours | 2.43 ± 0.98 | 2(2,3) | 3.51 ± 2.31 | 3(1,6) | 3.6 ± 1.66 | 4(2,5) |  |  |
| 2~3 hours | 2.34 ± 0.88 | 2(2,3) | 3.33 ± 2.21 | 3(1,5) | 3.33 ± 1.63 | 3(2,5) |  |  |
| More than 3 hours | 2.43 ± 0.96 | 2(2,3) | 3.69 ± 2.31 | 3(1,6) | 3.57 ± 1.69 | 4(2,5) |  |  |

*Note:*

*“1” means “To guide students to discover, propose and solve problems independently, and to stimulate students' interest and initiative in learning”.*

*“2” means “Provide real cases for students to analyze and solve problems in simulated real situations”.*

*“3” means “Divide students into groups to discuss different scenarios of acute sports injuries”.*

*“4” means “Provide simulated sports injury scenarios for students to perform first aid and disposal operations in a controlled environment”.*

*“5” means “Guide students to investigate information and find solutions independently when they encounter acute sports injury problems, and present and discuss them”.*

*“6” means “Combine the online platform and teaching resources to provide supplementary teaching resources such as course content, case studies and interactive discussions”.*

*“7” means “Organize students into teams to work together on the prevention and treatment of acute sports injuries after class, and finally present the team's research results”.*

Table S9 Number of People with Each Option by Subgroup

| Subgroup | 1 | 2 | 3 | 4 | 5 | 6 |
| --- | --- | --- | --- | --- | --- | --- |
| Male | 650 | 992 | 760 | 1042 | 867 | 1112 |
| Female | 666 | 958 | 792 | 1084 | 898 | 1138 |
| Northeast China | 98 | 165 | 108 | 163 | 135 | 179 |
| North China | 94 | 134 | 100 | 150 | 135 | 158 |
| East China | 688 | 1055 | 853 | 1144 | 954 | 1227 |
| South Central China | 272 | 365 | 311 | 414 | 338 | 429 |
| Southwest China | 81 | 118 | 86 | 132 | 95 | 125 |
| Northwest China | 83 | 113 | 94 | 123 | 108 | 132 |
| Grade 1 | 334 | 446 | 365 | 477 | 405 | 537 |
| Grade 2 | 410 | 589 | 497 | 658 | 537 | 672 |
| Grade 3 | 348 | 560 | 411 | 576 | 513 | 623 |
| Grade 4 | 167 | 270 | 205 | 312 | 224 | 326 |
| Grade 5 | 57 | 85 | 74 | 103 | 86 | 92 |
| Project 985 University | 361 | 413 | 353 | 412 | 373 | 433 |
| Project 211 University | 171 | 267 | 206 | 272 | 246 | 284 |
| State University | 530 | 825 | 650 | 940 | 761 | 1022 |
| Private University | 254 | 445 | 343 | 502 | 385 | 511 |
| Less than 2 hours | 740 | 1039 | 858 | 1176 | 973 | 1263 |
| 2~4 hours | 353 | 559 | 453 | 562 | 480 | 631 |
| 4~6 hours | 145 | 225 | 160 | 253 | 212 | 224 |
| More than 6hours | 78 | 127 | 81 | 135 | 100 | 132 |
| Less than 1 hour | 479 | 600 | 527 | 674 | 584 | 710 |
| 1~2 hours | 397 | 647 | 473 | 664 | 531 | 720 |
| 2~3 hours | 298 | 479 | 382 | 557 | 460 | 564 |
| More than 3 hours | 142 | 224 | 170 | 231 | 190 | 256 |

*Note: “1” means “Indicators related to the risk of cardiovascular events during exercise”; “2” means “Content of physical fitness tests”; “3” means “Content of pre-exercise warm-up or preparatory activities and post-exercise finishing activities and stretching exercises”; “4” means “Measures to be taken in case of discomfort”; “5” means “Proper exercise techniques for resistance training”; “6” means “Application of the RICE principle”.*

Table S10 Number of People with Each Option by Subgroup

| Subgroup | 1 | 2 | 3 | 4 | 5 |
| --- | --- | --- | --- | --- | --- |
| Male | 755 | 956 | 1106 | 1179 | 1021 |
| Female | 798 | 923 | 1129 | 1149 | 1053 |
| Northeast China | 109 | 141 | 200 | 207 | 172 |
| North China | 119 | 128 | 142 | 158 | 141 |
| East China | 860 | 1060 | 1269 | 1323 | 1145 |
| South Central China | 281 | 329 | 377 | 404 | 371 |
| Southwest China | 93 | 108 | 119 | 115 | 109 |
| Northwest China | 91 | 113 | 128 | 121 | 136 |
| Grade 1 | 374 | 425 | 461 | 496 | 470 |
| Grade 2 | 472 | 576 | 668 | 699 | 625 |
| Grade 3 | 426 | 550 | 686 | 702 | 602 |
| Grade 4 | 208 | 253 | 328 | 317 | 286 |
| Grade 5 | 73 | 75 | 92 | 114 | 91 |
| Project 985 University | 291 | 344 | 320 | 304 | 380 |
| Project 211 University | 213 | 252 | 318 | 329 | 285 |
| State University | 715 | 853 | 1041 | 1097 | 916 |
| Private University | 334 | 430 | 556 | 598 | 493 |
| Less than 2 hours | 845 | 1047 | 1213 | 1260 | 1117 |
| 2~4 hours | 422 | 495 | 639 | 668 | 591 |
| 4~6 hours | 185 | 225 | 250 | 260 | 236 |
| More than 6hours | 101 | 112 | 133 | 140 | 130 |
| Less than 1 hour | 491 | 582 | 602 | 611 | 623 |
| 1~2 hours | 481 | 613 | 766 | 800 | 681 |
| 2~3 hours | 414 | 487 | 610 | 648 | 521 |
| More than 3 hours | 167 | 197 | 257 | 269 | 249 |

*Note: “1” means “Lectures”; “2” means “Coursework organized by the school”; “3” means “Related knowledge brochures”; “4” means “Relevant books and literature”; “5” means “Internet channels (public number, WeChat, Netease Open Class, etc.)”.*

Table S11 Number of People with Each Option by Subgroup

| Subgroup | 1 | 2 | 3 | 4 | 5 | 6 | 7 |
| --- | --- | --- | --- | --- | --- | --- | --- |
| Male | 948 | 595 | 678 | 816 | 856 | 754 | 623 |
| Female | 1022 | 568 | 718 | 840 | 853 | 736 | 631 |
| Northeast China | 150 | 83 | 105 | 132 | 160 | 120 | 89 |
| North China | 138 | 76 | 92 | 114 | 107 | 111 | 82 |
| East China | 1084 | 584 | 771 | 956 | 985 | 821 | 676 |
| South Central China | 392 | 252 | 276 | 266 | 272 | 254 | 275 |
| Southwest China | 104 | 82 | 74 | 91 | 81 | 85 | 73 |
| Northwest China | 102 | 86 | 78 | 97 | 104 | 99 | 59 |
| Grade 1 | 452 | 300 | 358 | 351 | 359 | 351 | 293 |
| Grade 2 | 582 | 374 | 412 | 492 | 507 | 435 | 418 |
| Grade 3 | 571 | 273 | 382 | 494 | 531 | 429 | 332 |
| Grade 4 | 284 | 158 | 175 | 238 | 247 | 219 | 157 |
| Grade 5 | 81 | 58 | 69 | 81 | 65 | 56 | 54 |
| Project 985 University | 418 | 371 | 270 | 261 | 208 | 258 | 256 |
| Project 211 University | 262 | 141 | 186 | 241 | 240 | 194 | 157 |
| State University | 857 | 433 | 603 | 752 | 845 | 688 | 571 |
| Private University | 433 | 218 | 337 | 402 | 416 | 350 | 270 |
| Less than 2 hours | 1083 | 648 | 764 | 895 | 925 | 796 | 684 |
| 2~4 hours | 547 | 318 | 393 | 456 | 478 | 449 | 340 |
| 4~6 hours | 222 | 129 | 158 | 211 | 204 | 142 | 142 |
| More than 6hours | 118 | 68 | 81 | 94 | 102 | 103 | 88 |
| Less than 1 hour | 679 | 472 | 427 | 469 | 436 | 429 | 424 |
| 1~2 hours | 608 | 325 | 478 | 543 | 579 | 487 | 384 |
| 2~3 hours | 472 | 260 | 338 | 469 | 492 | 404 | 301 |
| More than 3 hours | 211 | 106 | 153 | 175 | 202 | 170 | 145 |

*Note: “1” means “School Campus”; “2” means “Public sports grounds”; “3” means “Hospitals”; “4” means “Neighborhoods”; “5” means “Libraries ”; “6” means “Parks”; “7” means “Online Platform”.*
